# Relationship between bullous pemphigoid and malignancy: A Mendelian randomization study

**DOI:** 10.1111/1346-8138.17100

**Published:** 2024-01-11

**Authors:** Ming‐Jie He, Yu‐Jia Wang, De‐Long Ran, De‐Shuang Fu, Qing He, Han‐Yin Zhang, Yu Mao, Peng‐Yuan Zhao, Jian‐Bin Yu, Jiang‐An Zhang

**Affiliations:** ^1^ Department of Dermatology First Affiliated Hospital of Zhengzhou University Zhengzhou Henan China; ^2^ Department of Cardiology First Affiliated Hospital of Zhengzhou University Zhengzhou Henan China

**Keywords:** bullous pemphigoid, causality, Mendelian randomization, neoplasms

## Abstract

Bullous pemphigoid (BP) is the most common autoimmune blistering disease, which primarily affects the elderly. However, the relationship between BP and malignancy remains controversial in traditional observational studies. The aim of this study, which included only European populations, was to assess the potential causative link between BP and 13 types of malignant tumors in a two‐sample Mendelian randomization (MR) study. BP was not associated with an increased risk of developing 13 types of malignant tumors. This study did not find a causal relationship between BP and malignant tumors. However, further research is warranted to examine the generalizability of this conclusion in non‐European populations.

## 1 INTRODUCTION

Bullous pemphigoid (BP) is a chronic autoimmune bullous disorder that primarily affects the elderly population. It is characterized by the formation of large, tense blisters on the skin and mucous membranes.^1^ The cumulative incidence of BP is 8.2 per 1 million people.^2^ BP is induced by autoantibodies targeting two hemidesmosomal proteins, namely BP180 (also known as BP antigen 2 or collagen XVII) and BP230 (also known as BP antigen 1).^3^

Cancer is expected to become the leading cause of death worldwide in the 21st century, as the global cancer burden continues to increase.^4^ The association between BP and malignancy remains controversial. A retrospective cohort study conducted in China revealed an increased predisposition to cancer among patients diagnosed with BP.^5^ A cohort study conducted in Germany demonstrated an increased prevalence of hematological tumors in individuals with BP in contrast to a healthy control group.^6^ Nonetheless, another retrospective cohort study found no statistical difference in the incidence of malignant tumors between the BP group and the control group, where the control group comprised age and gender matched individuals with other skin conditions, including psoriasis, lichen simplex chronicus, and xerosis.^7^ A systematic review and meta‐analysis also found no discernible correlation between BP and the occurrence of cancer across all study designs.^8^ In contrast, another meta‐analysis proposed an increased occurrence of malignancy among patients with BP in comparison with the control group.^9^ Some theories propose that antibodies targeting tumor‐specific antigens in malignant cells may cross‐react with basement membrane region antigens, including BP antigens, resulting in blister formation.^10^ Meanwhile, certain glucocorticoids and immunosuppressants used in the treatment of BP may potentially contribute to tumor development. Thus far, the correlation between BP and the susceptibility to malignancy has solely relied on observational studies, which are susceptible to confounding factors and the reversal of cause‐and‐effect associations. Hence, the causal relationship between the two has not been definitively established.

Mendelian randomization (MR) is a genetic epidemiological research methodology that employs single nucleotide polymorphisms (SNPs) as instrumental variables (IVs) to discern potential causal relationships between exposure factors and outcomes based on Mendelian laws of heredity.^11^, ^12^, ^13^ In contrast to observational studies, MR studies possess several advantages. As genetic variation is determined at the time of conception, it predates disease development and is generally unaffected by confounding factors, such as acquired factors and the social environment. Consequently, the inferred causal associations from MR studies exhibit more cogent temporality, diminish confounding bias, and mitigate reverse causation.^14^ This study utilized a two‐sample MR study design to explore the potential causal relationship between BP and malignant tumors, using the publicly available extensive genome‐wide association study (GWAS) database.^15^

## 2 MATERIALS AND METHODS

### 2.1 Study design

**FIGURE 1:**
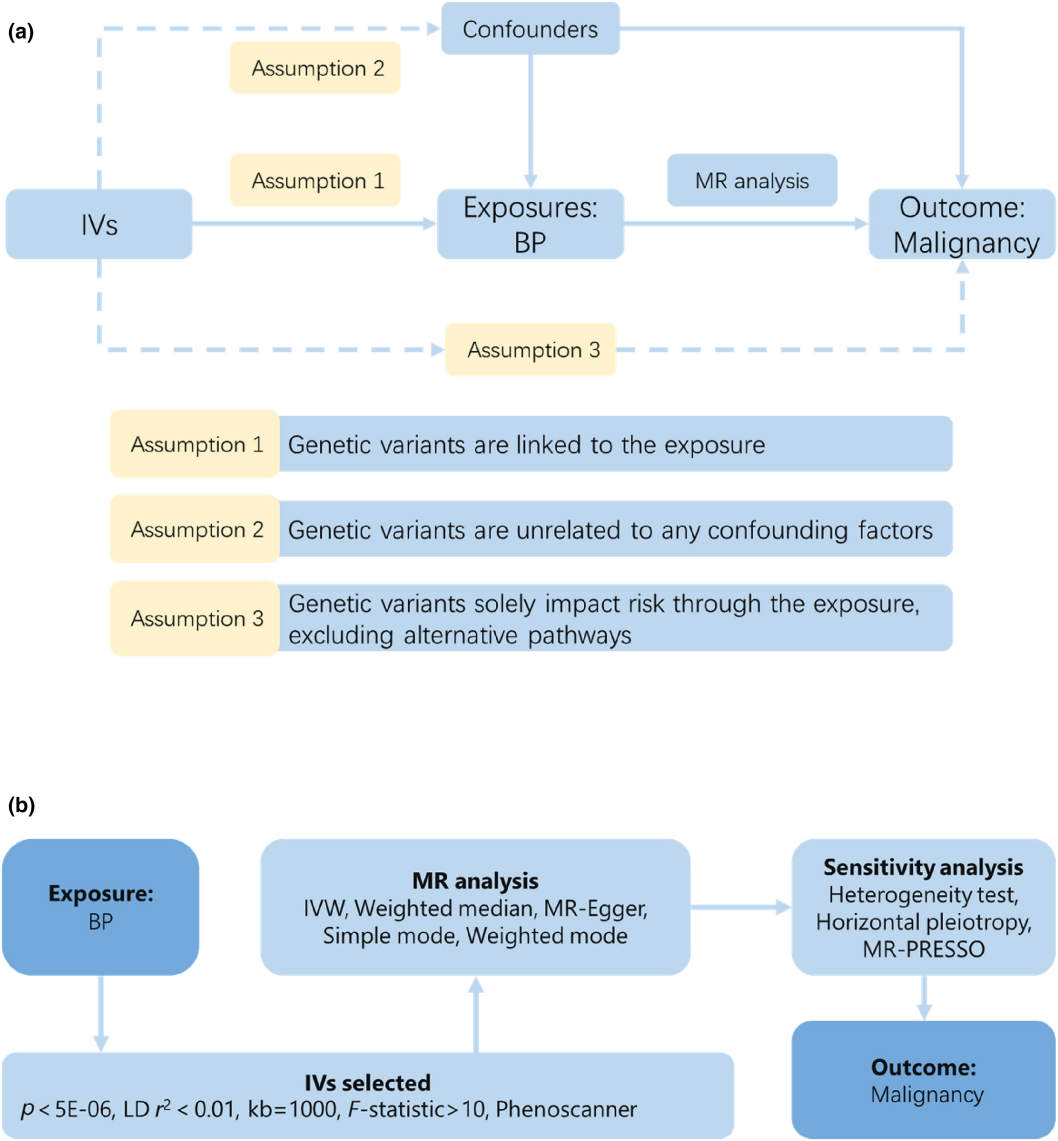
Study design overview. (a) Mendelian randomization (MR) analyses depend on three core assumptions.^17^ (b) Outline of the study design. BP, bullous pemphigoid; IVs, instrumental variables; IVW, inverse variance weighted; LD, linkage disequilibrium.

To ascertain a causal relationship between BP and malignancy, a two‐sample MR analysis was conducted utilizing a combined dataset sourced from GWAS.^16^ The data utilized in this investigation were acquired from previously published studies, which received ethical approval from their respective committees, thereby obviating the need for additional ethical clearance. The pictorial representation of the study design is shown in Figure 1.^15^

### 2.2 Data sources

To conduct MR analyses, we utilized summary‐level data obtained from the publicly available GWAS database. We used GWAS with European descent as the data source of genetic instruments. Genetic IVs for BP (*n* = 218 285) were obtained from GWAS in the FinnGen (https://www.finngen.fi/en). Genetic IVs for malignant tumors were all obtained from GWAS in the UK biobank (*n* = 442 239; Table S1).

### 2.3 Genetic IV selection

IV selection criteria in this study:

1. SNPs exhibiting a genome‐wide significant association with the BP phenotype (*p* < 5E‐06).^18^
2. Lack of linkage disequilibrium (LD) between SNPs. Quality control standard: *r* ^2^ < 0.01, kb = 1000.^19^
3. Integration and concordance of the exposure‐outcome dataset, along with correction of palindromic SNPs with an ambiguous strand based on allele frequency information.^20^
4. Screening of SNPs selected through the previous steps in the PhenoScanner database (www.phenoscanner.medschl.cam.ac.uk/)^21^ to identify variants linked to other phenotypes below the threshold of *p* < 5E‐08, which could potentially influence the outcome progression independently of the exposure.^19^
5. Evaluation of IV strength by calculating the *F*‐value, and exclusion of potentially weak IV bias between the IV and exposure factors (*F* > 10).^19^, ^22^ The formula for calculating *F*‐value is shown in Appendix S1.

### 2.4 Statistical analysis

In this study, the “Two‐Sample MR” and “MRPRESSO” packages of R 4.1.0 software were utilized for analysis. We primarily utilized the inverse variance weighted (IVW) method^23^ to compute the odds ratio (OR) and its 95% confidence interval (CI) to assess the potential causal relationship between BP and malignant tumors. The other four methods were the MR‐Egger regression method,^24^ weighted median method (WME),^25^ weighted model, and simple model. Given the susceptibility of IVW estimates to invalid instrumental bias or pleiotropy, a series of sensitivity analyses were conducted to ascertain the validity and robustness of the IVW results. The assessment of heterogeneity among SNPs was performed using Cochran's Q.^26^ Significance was indicated by a *p* value of < 0.05, leading to the adoption of a random effects model. Otherwise, a fixed effects model was employed. To assess horizontal pleiotropy, we utilized a funnel plot and applied the MR‐Egger method.^11^, ^12^, ^13^, ^27^ To identify any potential outlier instruments contributing to horizontal pleiotropy, we employed the MR‐PRESSO (MR pleiotropy residual sum and outlier) method, with a significance threshold of *p* < 0.05. Outliers were identified and subsequently removed in a stepwise manner to minimize the impact of horizontal pleiotropy. Furthermore, the leave‐one‐out method^28^ was utilized to identify the IVs that could potentially impact the MR results. To address the issue of multiple testing, the Benjamini‐Hochberg method was employed, which incorporates the false discovery rate.^29^ All statistical tests employed bilateral analysis, and a *p* value of < 0.05 was deemed statistically significant.

## 3 RESULTS

### 3.1 The outcomes of IV selection

There were 14 SNPs serving as IVs for BP, following LD adjustments, at the GWAS threshold of *p* < 5E‐06. Certain SNPs were excluded due to their association with confounding factors or possession of palindromic sequences. The absence of weak IV bias was confirmed as all IVs demonstrated significant *F*‐values exceeding 10, including rs9996810 (*F* = 21.0174), rs79334883 (*F* = 21.5786), rs76965871 (*F* = 24.7926), rs75308030 (*F* = 24.8402), rs60559879 (*F* = 21.2687), rs35604167 (*F* = 23.7677), rs3094169 (*F* = 21.7826), rs200148 (*F* = 23.4571), rs144298683 (*F* = 28.2940), and rs10483424 (*F* = 24.9983). The detailed information is listed in Tables S2–S14. The effects of a single SNP on malignancy are shown in Figure S1 (scatter plot) and Figure S2 (forest plot).

### 3.2 Effect of bullous pemphigoid on 13 malignancies

**FIGURE 2:**
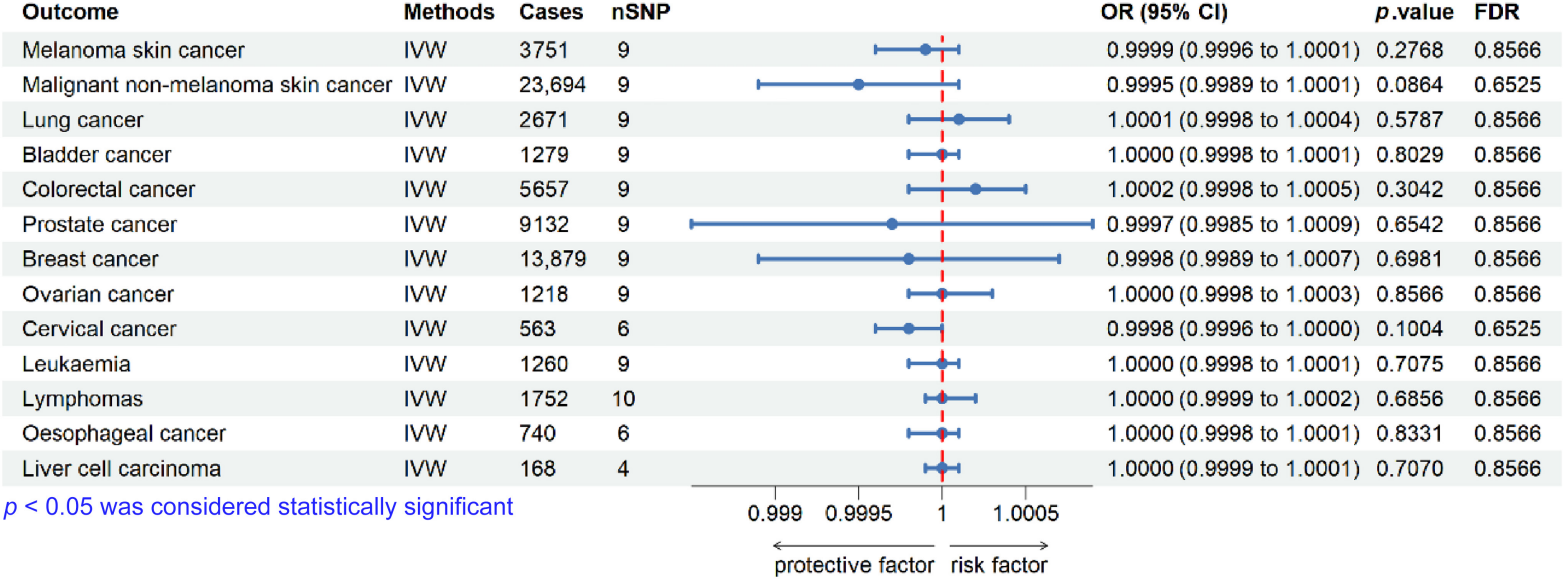
Relationship between bullous pemphigoid (BP) and 13 malignancies in Mendelian randomization. The causal estimates are presented as odds ratios (ORs) along with their corresponding 95% confidence intervals (CIs). False discovery rate (FDR), the adjusted *p*‐value by the Benjamini–Hochberg method. IVW, inverse variance weighted.

The inverse variance weighted median method, simple model, weighted model, and MR‐Egger regression were used to estimate the causal relationship between genetically predicted BP and the risk of developing malignancy. After applying the Benjamini‐Hochberg adjustment, the MR analysis revealed no significant correlation between genetically predicted BP and 13 types of malignant tumors. Figure 2 depicts the outcomes obtained through the IVW approach, while the detailed outcomes from all methods can be found in Table S15.

### 3.3 Sensitivity analysis

We observed heterogeneity in the causal effects of SNPs for lung cancer and prostate cancer (Cochran's Q, *p* < 0.05). Consequently, we employed a random‐effects IVW approach to mitigate this issue. However, even after applying this method, the resulting *p* value remained above 0.05, indicating no significant association between BP and the occurrence of these two types of tumors (Table S16). Both the funnel plots (Figure S3) and MR‐Egger test (Table S17) did not indicate substantial horizontal pleiotropy, suggesting the absence of significant pleiotropic effects in this study. During the sensitivity analysis conducted using the MR‐PRESSO method, a solitary outlier (rs10483424) was detected, solely in relation to the outcome of prostate cancer. After excluding this outlier, the resulting *p* value of 0.58 did not suggest a causal relationship between BP and prostate cancer. No outliers were detected during the sensitivity analysis conducted on the other 12 malignancies. We also conducted the leave‐one‐out method to identify the effect of each SNP on the overall causal estimates. No significant differences were observed in the estimated causal effects (Figure S4).

## 4 DISCUSSION

This was the first study to assess a causal relationship between BP and malignant tumors through a MR approach. Using publicly available BP and malignant tumor GWAS data, we systematically scrutinized the genetic associations between BP prediction and malignant tumors. After Benjamini–Hochberg correction, no causal link was found between the genetic susceptibility of BP and 13 distinct malignant tumors. There are areas of agreement or contradiction between our conclusions and those of previous observational studies.

In a retrospective cohort study,^7^ which included 99 BP patients from two hospitals, no significant association was found between BP and malignancy. The rates of malignancy did not significantly differ between BP patients and controls (OR 0.86, 95% CI  0.47–1.58). This conclusion is consistent with our findings. In a systematic review and meta‐analysis of eight studies,^8^ no association was found between BP and overall malignancy (OR 2.04, 95% CI 0.97–4.31), but a possible association was observed between BP and hematological malignancy (OR 2.60, 95% CI  2.08–3.24). Several retrospective studies reported that BP is not associated with overall malignancy, but with specific malignancies. This conflicts with the conclusions of our study. For example, Baum et al.^30^ reported a positive correlation between BP and an increased susceptibility to melanoma. Schulze et al.^6^ noted that patients with BP exhibited an increased risk of hematological malignancies (OR 2.55, 95% CI  2.07–3.13). Our findings indicated no causal relationship between BP and malignant tumors. Some of the previous observational studies may have been confounded by potential covariates, such as age. As BP is more prevalent in the elderly, the incidence of malignancies is higher in this demographic. Two supporting studies bolster this perspective. Lindelöf et al.^31^ observed no significant difference in circulating anti‐basement membrane antibody titers between BP patients with and without malignancies. They also calculated the expected number of malignancies in BP patients based on age‐ and sex‐standardized incidence data, finding no significant disparity compared with observed cases. Similarly, in a parallel study, Cai et al.^32^ compared expected cancer cases in the general population with actual occurrences in BP patients (matched for sex and age), revealing no significant difference. Another potential confounding factor could be the use of glucocorticoids or immunosuppressants.^33^, ^34^ Therefore, a prospective study is needed to control for these potential confounding factors.

The primary strength of this study lies in its pioneering use of MR methods to assess the causal relationship between genetically predicted BP and the risk of 13 types of malignancies. Given that allele segregation during conception is randomized and fixed, this study avoided biases arising from confounding factors and reverse causation, which have been limitations in previous observational studies.^35^ However, this study also had some limitations. First, public databases currently only contain GWAS data for BP in European populations, limiting our ability to explore potential differences between European and East Asian populations. This limitation underscores the necessity for future studies to include diverse populations to unravel a more comprehensive understanding of the relationship between BP and malignancy. Second, due to the absence of detailed clinical data, subgroup analyses were not feasible. Third, despite utilizing the PhenoScanner website to retrieve potential confounding factors, it is important to acknowledge that there may be undiscovered confounding factors without corresponding SNP data, making their exclusion through PhenoScanner challenging and potentially leading to biased results.

In summary, no causal relationship was found between BP and malignancies in this study, which could potentially alleviate anxiety among individuals with BP and reduce unnecessary cancer screenings.

## FUNDING INFORMATION

This research received no external funding.

## CONFLICT OF INTEREST STATEMENT

The authors have no conflicts of interest to declare.

## Supporting information


Appendix S1.

## Data Availability

All summary statistics data used in this work were from genome‐wide association studies and are publicly available at https://gwas.mrcieu.ac.uk/.
