# Supplementary material for: Relationship between bullous pemphigoid and malignancy: A Mendelian randomization study
Source: J Dermatol. 2024 Jan 11;51(3):403–8. doi: 10.1111/1346-8138.17100 (PMC11483921; doi:10.1111/1346-8138.17100)
Supplement: Supplementary file 1 — Appendix S1. [file JDE-51--s001.docx]

**Supplement contents**

**Supplementary figures and tables**

| \| **Supplementary Table S1.**  Details of the data sources used in this study. \| \| --- \| | | | | | | |
| --- | --- | --- | --- | --- | --- | --- | --- |
| **Factors** | **Consortium** | **Samples** | **Cases** | **Controls** | **Population** | **Source** |
| Bullous pemphigoid | FinnGen Biobank | 218,285 | 219 | 218,066 | European | https://gwas.mrcieu.ac.uk/datasets/finn-b-L12_PEMPHIGOID/ |
| Melanoma skin cancer | UK Biobank | 375,767 | 3,751 | 372,016 | European | https://gwas.mrcieu.ac.uk/datasets/ieu-b-4969/ |
| Malignant non-melanoma skin cancer | UK Biobank | 395,710 | 23,694 | 372,016 | European | https://gwas.mrcieu.ac.uk/datasets/ieu-b-4959/ |
| Lung cancer | UK Biobank | 374,687 | 2,671 | 372,016 | European | https://gwas.mrcieu.ac.uk/datasets/ieu-b-4954/ |
| Bladder cancer | UK Biobank | 373,295 | 1,279 | 372,016 | European | https://gwas.mrcieu.ac.uk/datasets/ieu-b-4874/ |
| Colorectal cancer | UK Biobank | 377,673 | 5,657 | 372,016 | European | https://gwas.mrcieu.ac.uk/datasets/ieu-b-4965/ |
| Prostate cancer | UK Biobank | 182,625 | 9,132 | 173,493 | European | https://gwas.mrcieu.ac.uk/datasets/ieu-b-4809/ |
| Breast cancer | UK Biobank | 198,523 | 13,879 | 198,523 | European | https://gwas.mrcieu.ac.uk/datasets/ieu-b-4810/ |
| Ovarian cancer | UK Biobank | 199,741 | 1,218 | 198,523 | European | https://gwas.mrcieu.ac.uk/datasets/ieu-b-4963/ |
| Cervical cancer | UK Biobank | 199,086 | 563 | 198,523 | European | https://gwas.mrcieu.ac.uk/datasets/ieu-b-4876/ |
| Leukaemia | UK Biobank | 373,276 | 1,260 | 372,016 | European | https://gwas.mrcieu.ac.uk/datasets/ieu-b-4914/ |
| Lymphomas | UK Biobank | 361,194 | 1,752 | 359,442 | European | https://gwas.mrcieu.ac.uk/datasets/ukb-d-C_LYMPHOMA/ |
| Oesophageal cancer | UK Biobank | 372,756 | 740 | 372,016 | European | https://gwas.mrcieu.ac.uk/datasets/ieu-b-4960/ |
| Liver cell carcinoma | UK Biobank | 372,184 | 168 | 372,016 | European | https://gwas.mrcieu.ac.uk/datasets/ieu-b-4953/ |

| **Supplementary Table S2.**  SNPs utilized as instruments for BP in final MR analysis on melanoma skin cancer. | | | | | | | | | | | | | | | | | |  |
| --- | --- | --- | --- | --- | --- | --- | --- | --- | --- | --- | --- | --- | --- | --- | --- | --- | --- | --- |
|  | BP | | | | | | | | | | | Melanoma skin cancer | | | | | | |
|  | **SNP** | **CHR** | **POS** | **MAF** | **EA** | **OA** | **beta** | **eaf** | **pval** | **se** | **samplesize** | **beta** | **eaf** | **se** | **pval** | **samplesize** | **R^2^** | **F** |
| 1 | rs9996810 | 4 | 62468670 | 0.2406 | C | T | 0.5263 | 0.2406 | 4.52E-06 | 0.1148 | 218285 | 0.0002 | 0.2179 | 0.0003 | 0.5200 | 375767 | 9.6276E-05 | 21.0174 |
| 2 | rs79334883 | 4 | 86211322 | 0.0421 | G | A | 1.2389 | 0.0421 | 3.38E-06 | 0.2667 | 218285 | -0.0002 | 0.0287 | 0.0007 | 0.7600 | 375767 | 9.8846E-05 | 21.5786 |
| 3 | rs7696587 | 1 | 175729422 | 0.0111 | A | G | 3.0219 | 0.0111 | 6.37E-07 | 0.6069 | 218285 | -0.0011 | 0.0269 | 0.0007 | 0.1300 | 375767 | 1.1357E-04 | 24.7926 |
| 4 | rs75308030 | 10 | 26019862 | 0.0063 | T | G | 4.7413 | 0.0063 | 6.23E-07 | 0.9513 | 218285 | 0.0004 | 0.0115 | 0.0011 | 0.7100 | 375767 | 1.1379E-04 | 24.8403 |
| 5 | rs60559879 | 17 | 35192062 | 0.2705 | A | G | 0.5073 | 0.2705 | 3.99E-06 | 0.1100 | 218285 | -0.0001 | 0.2813 | 0.0003 | 0.7400 | 375767 | 9.7427E-05 | 21.2687 |
| 6 | rs35604167 | 4 | 169382451 | 0.1114 | T | G | 0.7932 | 0.1114 | 1.09E-06 | 0.1627 | 218285 | 0.0001 | 0.1241 | 0.0004 | 0.6900 | 375767 | 1.0887E-04 | 23.7677 |
| 7 | rs10483424 | 14 | 33591131 | 0.0089 | A | G | 3.3364 | 0.0089 | 5.73E-07 | 0.6673 | 218285 | -0.0014 | 0.0165 | 0.0009 | 0.1100 | 375767 | 1.1451E-04 | 24.9983 |
| 8 | rs144298683 | 3 | 88344440 | 0.0139 | T | C | 2.7543 | 0.0139 | 1.05E-07 | 0.5178 | 218285 | 0.0001 | 0.0105 | 0.0011 | 0.9100 | 375767 | 1.2960E-04 | 28.2940 |
| 9 | rs3094169 | 6 | 29829986 | 0.2806 | C | T | -0.5736 | 0.7194 | 3.04E-06 | 0.1229 | 218285 | -0.00001 | 0.8098 | 0.0002 | 0.9200 | 375767 | 9.9781E-05 | 21.7826 |

Abbreviations: BP, Bullous pemphigoid; CHR, chromosome; MAF, minor allele frequency; MR, Mendelian randomization; POS, position; SNPs, single‐nucleotide polymorphisms.

Note(a): 14 independent genome wide significant SNPs were extracted from BP GWAS (P < 5E-06; LD R^2^ < 0.01, 1000kb); 10 of these instruments were found in Melanoma skin cancer GWAS; 3 SNPs (rs145834662, rs147633772, rs11205458) were removed for being palindromic with intermediate allele frequencies (harmonization).

Note(b): At the more stringent threshold of P < 5E-08, No SNPs linked with other phenotypes were identified on the PhenoScancer website.

Note(c): $R^{2}=\frac{2\times\beta^{2}\times EAF\times(1-EAF)}{2\times\beta^{2}\times EAF\times\left( 1-EAF \right)+2\times{SE}^{2}\times N\times EAF\times(1-EAF)}$ ,$F=\frac{R^{2}\times(N-2)}{1-R^{2}}$. N represents the number of participants, EAF represents the effect allele frequency, and β is the estimated effect of the SNP to assess its ability to uniquely predict the outcome.

| **Supplementary Table S3.**  SNPs utilized as instruments for BP in final MR analysis on Malignant non-melanoma skin cancer. | | | | | | | | | | | | | | | | | |  |
| --- | --- | --- | --- | --- | --- | --- | --- | --- | --- | --- | --- | --- | --- | --- | --- | --- | --- | --- |
|  | BP | | | | | | | | | | | Malignant non-melanoma skin cancer | | | | | | |
|  | **SNP** | **CHR** | **POS** | **MAF** | **EA** | **OA** | **beta** | **eaf** | **pval** | **se** | **samplesize** | **beta** | **eaf** | **se** | **pval** | **samplesize** | **R^2^** | **F** |
| 1 | rs9996810 | 4 | 62468670 | 0.2406 | C | T | 0.5263 | 0.2406 | 4.52E-06 | 0.1148 | 218285 | 0.0005 | 0.2180 | 0.0006 | 0.4700 | 395710 | 9.6276E-05 | 21.0174 |
| 2 | rs79334883 | 4 | 86211322 | 0.0421 | G | A | 1.2389 | 0.0421 | 3.38E-06 | 0.2667 | 218285 | 0.0011 | 0.0287 | 0.0016 | 0.5000 | 395710 | 9.8846E-05 | 21.5786 |
| 3 | rs76965871 | 1 | 175729422 | 0.0111 | A | G | 3.0219 | 0.0111 | 6.37E-07 | 0.6069 | 218285 | -0.0008 | 0.0269 | 0.0016 | 0.6500 | 395710 | 1.1357E-04 | 24.7926 |
| 4 | rs75308030 | 10 | 26019862 | 0.0063 | T | G | 4.7413 | 0.0063 | 6.23E-07 | 0.9513 | 218285 | -0.0063 | 0.0115 | 0.0027 | 0.0180 | 395710 | 1.1379E-04 | 24.8402 |
| 5 | rs60559879 | 17 | 35192062 | 0.2705 | A | G | 0.5073 | 0.2705 | 3.99E-06 | 0.1100 | 218285 | -0.0007 | 0.2812 | 0.0006 | 0.2100 | 395710 | 9.7427E-05 | 21.2687 |
| 6 | rs35604167 | 4 | 169382451 | 0.1114 | T | G | 0.7932 | 0.1114 | 1.09E-06 | 0.1627 | 218285 | 0.0008 | 0.1242 | 0.0008 | 0.3400 | 395710 | 1.0887E-04 | 23.7677 |
| 7 | rs10483424 | 14 | 33591131 | 0.0089 | A | G | 3.3364 | 0.0089 | 5.73E-07 | 0.6673 | 218285 | -0.0038 | 0.0165 | 0.0021 | 0.0710 | 395710 | 1.1451E-04 | 24.9983 |
| 8 | rs144298683 | 3 | 88344440 | 0.0139 | T | C | 2.7543 | 0.0139 | 1.05E-07 | 0.5178 | 218285 | 0.0017 | 0.0105 | 0.0026 | 0.5300 | 395710 | 1.2960E-04 | 28.2940 |
| 9 | rs3094169 | 6 | 29829986 | 0.2806 | C | T | -0.5736 | 0.7194 | 3.04E-06 | 0.1229 | 218285 | 0.0008 | 0.8100 | 0.0007 | 0.2300 | 395710 | 9.9781E-05 | 21.7826 |

Abbreviations: BP, Bullous pemphigoid; CHR, chromosome; MAF, minor allele frequency; MR, Mendelian randomization; POS, position; SNPs, single‐nucleotide polymorphisms.

Note(a): 14 independent genome wide significant SNPs were extracted from BP GWAS (P < 5E-06; LD R^2^ < 0.01, 1000kb); 10 of these instruments were found in Malignant non-melanoma skin cancer GWAS; 3 SNPs (rs145834662, rs147633772, rs11205458) were removed for being palindromic with intermediate allele frequencies (harmonization).

Note(b): At the more stringent threshold of P < 5E-08, No SNPs linked with other phenotypes were identified on the PhenoScancer website.

Note(c): $R^{2}=\frac{2\times\beta^{2}\times EAF\times(1-EAF)}{2\times\beta^{2}\times EAF\times\left( 1-EAF \right)+2\times{SE}^{2}\times N\times EAF\times(1-EAF)}$ ,$F=\frac{R^{2}\times(N-2)}{1-R^{2}}$. N = GWAS sample size; R^2^ is the proportion of variation in the exposure database explained by SNPs; β= effect estimate; EAF=effect allele frequency.

| **Supplementary Table S4.**  SNPs utilized as instruments for BP in final MR analysis on Lung cancer. | | | | | | | | | | | | | | | | | |  |
| --- | --- | --- | --- | --- | --- | --- | --- | --- | --- | --- | --- | --- | --- | --- | --- | --- | --- | --- |
|  | BP | | | | | | | | | | | Lung cancer | | | | | | |
|  | **SNP** | **CHR** | **POS** | **MAF** | **EA** | **OA** | **beta** | **eaf** | **pval** | **se** | **samplesize** | **beta** | **eaf** | **se** | **pval** | **samplesize** | **R^2^** | **F** |
| 1 | rs9996810 | 4 | 62468670 | 0.2406 | C | T | 0.5263 | 0.2406 | 4.52E-06 | 0.1148 | 218285 | -0.0005 | 0.2178 | 0.0002 | 0.0510 | 374687 | 9.6276E-05 | 21.0174 |
| 2 | rs79334883 | 4 | 86211322 | 0.0421 | G | A | 1.2389 | 0.0421 | 3.38E-06 | 0.2667 | 218285 | 0.0009 | 0.0287 | 0.0006 | 0.1300 | 374687 | 9.8846E-05 | 21.5786 |
| 3 | rs76965871 | 1 | 175729422 | 0.0111 | A | G | 3.0219 | 0.0111 | 6.37E-07 | 0.6069 | 218285 | 0.0013 | 0.0270 | 0.0006 | 0.0250 | 374687 | 1.1357E-04 | 24.7926 |
| 4 | rs75308030 | 10 | 26019862 | 0.0063 | T | G | 4.7413 | 0.0063 | 6.23E-07 | 0.9513 | 218285 | 0.0018 | 0.0115 | 0.0010 | 0.0630 | 374687 | 1.1379E-04 | 24.8402 |
| 5 | rs60559879 | 17 | 35192062 | 0.2705 | A | G | 0.5073 | 0.2705 | 3.99E-06 | 0.1100 | 218285 | -0.0003 | 0.2813 | 0.0002 | 0.2200 | 374687 | 9.7427E-05 | 21.2687 |
| 6 | rs35604167 | 4 | 169382451 | 0.1114 | T | G | 0.7932 | 0.1114 | 1.09E-06 | 0.1627 | 218285 | -0.0005 | 0.1240 | 0.0003 | 0.0800 | 374687 | 1.0887E-04 | 23.7677 |
| 7 | rs10483424 | 14 | 33591131 | 0.0089 | A | G | 3.3364 | 0.0089 | 5.73E-07 | 0.6673 | 218285 | 0.0001 | 0.0165 | 0.0008 | 0.9300 | 374687 | 1.1451E-04 | 24.9983 |
| 8 | rs144298683 | 3 | 88344440 | 0.0139 | T | C | 2.7543 | 0.0139 | 1.05E-07 | 0.5178 | 218285 | -0.0018 | 0.0105 | 0.0010 | 0.0670 | 374687 | 1.2960E-04 | 28.2940 |
| 9 | rs3094169 | 6 | 29829986 | 0.2806 | C | T | -0.5736 | 0.7194 | 3.04E-06 | 0.1229 | 218285 | -0.0003 | 0.8098 | 0.0002 | 0.2200 | 374687 | 9.9781e-05 | 21.7826 |

Abbreviations: BP, Bullous pemphigoid; CHR, chromosome; MAF, minor allele frequency; MR, Mendelian randomization; POS, position; SNPs, single‐nucleotide polymorphisms.

Note(a): 14 independent genome wide significant SNPs were extracted from BP GWAS (P < 5E-06; LD R^2^ < 0.01, 1000kb); 10 of these instruments were found in Lung cancer GWAS; 3 SNPs (rs145834662, rs147633772, rs11205458) were removed for being palindromic with intermediate allele frequencies (harmonization).

Note(b): At the more stringent threshold of P < 5E-08, No SNPs linked with other phenotypes were identified on the PhenoScancer website.

Note(c): $R^{2}=\frac{2\times\beta^{2}\times EAF\times(1-EAF)}{2\times\beta^{2}\times EAF\times\left( 1-EAF \right)+2\times{SE}^{2}\times N\times EAF\times(1-EAF)}$ ,$F=\frac{R^{2}\times(N-2)}{1-R^{2}}$. N = GWAS sample size; R^2^ is the proportion of variation in the exposure database explained by SNPs; β= effect estimate; EAF=effect allele frequency.

| **Supplementary Table S5.**  SNPs utilized as instruments for BP in final MR analysis on Bladder cancer. | | | | | | | | | | | | | | | | | |  |
| --- | --- | --- | --- | --- | --- | --- | --- | --- | --- | --- | --- | --- | --- | --- | --- | --- | --- | --- |
|  | BP | | | | | | | | | | | Bladder cancer | | | | | | |
|  | **SNP** | **CHR** | **POS** | **MAF** | **EA** | **OA** | **beta** | **eaf** | **pval** | **se** | **samplesize** | **beta** | **eaf** | **se** | **pval** | **samplesize** | **R^2^** | **F** |
| 1 | rs9996810 | 4 | 62468670 | 0.2406 | C | T | 0.5263 | 0.2406 | 4.52E-06 | 0.1148 | 218285 | 0.0001 | 0.2179 | 0.0002 | 0.6900 | 373295 | 9.6276E-05 | 21.0174 |
| 2 | rs79334883 | 4 | 86211322 | 0.0421 | G | A | 1.2389 | 0.0421 | 3.38E-06 | 0.2667 | 218285 | 0.0001 | 0.0287 | 0.0004 | 0.8200 | 373295 | 9.8846E-05 | 21.5786 |
| 3 | rs76965871 | 1 | 175729422 | 0.0111 | A | G | 3.0219 | 0.0111 | 6.37E-07 | 0.6069 | 218285 | -0.0003 | 0.0269 | 0.0004 | 0.4800 | 373295 | 1.1357E-04 | 24.7926 |
| 4 | rs75308030 | 10 | 26019862 | 0.0063 | T | G | 4.7413 | 0.0063 | 6.23E-07 | 0.9513 | 218285 | -0.0005 | 0.0115 | 0.0007 | 0.4900 | 373295 | 1.1379E-04 | 24.8402 |
| 5 | rs60559879 | 17 | 35192062 | 0.2705 | A | G | 0.5073 | 0.2705 | 3.99E-06 | 0.1100 | 218285 | 0.0004 | 0.2814 | 0.0002 | 0.0150 | 373295 | 9.7427E-05 | 21.2687 |
| 6 | rs35604167 | 4 | 169382451 | 0.1114 | T | G | 0.7932 | 0.1114 | 1.09E-06 | 0.1627 | 218285 | -0.0003 | 0.1241 | 0.0002 | 0.1600 | 373295 | 1.0887E-04 | 23.7677 |
| 7 | rs10483424 | 14 | 33591131 | 0.0089 | A | G | 3.3364 | 0.0089 | 5.73E-07 | 0.6673 | 218285 | 0.0003 | 0.0165 | 0.0005 | 0.5700 | 373295 | 1.1451E-04 | 24.9983 |
| 8 | rs144298683 | 3 | 88344440 | 0.0139 | T | C | 2.7543 | 0.0139 | 1.05E-07 | 0.5178 | 218285 | 0.0001 | 0.0105 | 0.0007 | 0.8600 | 373295 | 1.2960E-04 | 28.2940 |
| 9 | rs3094169 | 6 | 29829986 | 0.2806 | C | T | -0.5736 | 0.7194 | 3.04E-06 | 0.1229 | 218285 | -0.0001 | 0.8099 | 0.0002 | 0.6100 | 373295 | 9.9781E-05 | 21.7826 |

Abbreviations: BP, Bullous pemphigoid; CHR, chromosome; MAF, minor allele frequency; MR, Mendelian randomization; POS, position; SNPs, single‐nucleotide polymorphisms.

Note(a): 14 independent genome wide significant SNPs were extracted from BP GWAS (P < 5E-06; LD R^2^ < 0.01, 1000kb); 12 of these instruments were found in Bladder cancer GWAS; 3 SNPs (rs145834662, rs147633772, rs11205458) were removed for being palindromic with intermediate allele frequencies (harmonization).

Note(b): At the more stringent threshold of P < 5E-08, No SNPs linked with other phenotypes were identified on the PhenoScancer website.

Note(c): $R^{2}=\frac{2\times\beta^{2}\times EAF\times(1-EAF)}{2\times\beta^{2}\times EAF\times\left( 1-EAF \right)+2\times{SE}^{2}\times N\times EAF\times(1-EAF)}$ ,$F=\frac{R^{2}\times(N-2)}{1-R^{2}}$. N = GWAS sample size; R^2^ is the proportion of variation in the exposure database explained by SNPs; β= effect estimate; EAF=effect allele frequency.

| **Supplementary Table S6.**  SNPs utilized as instruments for BP in final MR analysis on Colorectal cancer. | | | | | | | | | | | | | | | | | |  |
| --- | --- | --- | --- | --- | --- | --- | --- | --- | --- | --- | --- | --- | --- | --- | --- | --- | --- | --- |
|  | BP | | | | | | | | | | | Colorectal cancer | | | | | | |
|  | **SNP** | **CHR** | **POS** | **MAF** | **EA** | **OA** | **beta** | **eaf** | **pval** | **se** | **samplesize** | **beta** | **eaf** | **se** | **pval** | **samplesize** | **R^2^** | **F** |
| 1 | rs9996810 | 4 | 62468670 | 0.2406 | C | T | 0.5263 | 0.2406 | 4.52E-06 | 0.1148 | 218285 | 0.0005 | 0.2180 | 0.0003 | 0.1100 | 377673 | 9.6276E-05 | 21.0174 |
| 2 | rs79334883 | 4 | 86211322 | 0.0421 | G | A | 1.2389 | 0.0421 | 3.38E-06 | 0.2667 | 218285 | 0.0012 | 0.0287 | 0.0008 | 0.1400 | 377673 | 9.8846E-05 | 21.5786 |
| 3 | rs76965871 | 1 | 175729422 | 0.0111 | A | G | 3.0219 | 0.0111 | 6.37E-07 | 0.6069 | 218285 | 0.0008 | 0.0270 | 0.0009 | 0.3800 | 377673 | 1.1357E-04 | 24.7926 |
| 4 | rs75308030 | 10 | 26019862 | 0.0063 | T | G | 4.7413 | 0.0063 | 6.23E-07 | 0.9513 | 218285 | 0.0009 | 0.0115 | 0.0014 | 0.5000 | 377673 | 1.1379E-04 | 24.8402 |
| 5 | rs60559879 | 17 | 35192062 | 0.2705 | A | G | 0.5073 | 0.2705 | 3.99E-06 | 0.1100 | 218285 | -0.0001 | 0.2813 | 0.0003 | 0.7400 | 377673 | 9.7427E-05 | 21.2687 |
| 6 | rs35604167 | 4 | 169382451 | 0.1114 | T | G | 0.7932 | 0.1114 | 1.09E-06 | 0.1627 | 218285 | -0.0004 | 0.1240 | 0.0004 | 0.3200 | 377673 | 1.0887E-04 | 23.7677 |
| 7 | rs10483424 | 14 | 33591131 | 0.0089 | A | G | 3.3364 | 0.0089 | 5.73E-07 | 0.6673 | 218285 | 0.0023 | 0.0166 | 0.0011 | 0.0400 | 377673 | 1.1451E-04 | 24.9983 |
| 8 | rs144298683 | 3 | 88344440 | 0.0139 | T | C | 2.7543 | 0.0139 | 1.05E-07 | 0.5178 | 218285 | -0.0011 | 0.0105 | 0.0014 | 0.4300 | 377673 | 1.2960E-04 | 28.2940 |
| 9 | rs3094169 | 6 | 29829986 | 0.2806 | C | T | -0.5736 | 0.7194 | 3.04E-06 | 0.1229 | 218285 | 0.0006 | 0.8100 | 0.0004 | 0.0710 | 377673 | 9.9781E-05 | 21.7826 |

Abbreviations: BP, Bullous pemphigoid; CHR, chromosome; MAF, minor allele frequency; MR, Mendelian randomization; POS, position; SNPs, single‐nucleotide polymorphisms.

Note(a): 14 independent genome wide significant SNPs were extracted from BP GWAS (P < 5E-06; LD R^2^ < 0.01, 1000kb); 12 of these instruments were found in Colorectal cancer GWAS; 3 SNPs (rs145834662, rs147633772, rs11205458) were removed for being palindromic with intermediate allele frequencies (harmonization).

Note(b): At the more stringent threshold of P < 5E-08, No SNPs linked with other phenotypes were identified on the PhenoScancer website.

Note(c): $R^{2}=\frac{2\times\beta^{2}\times EAF\times(1-EAF)}{2\times\beta^{2}\times EAF\times\left( 1-EAF \right)+2\times{SE}^{2}\times N\times EAF\times(1-EAF)}$ ,$F=\frac{R^{2}\times(N-2)}{1-R^{2}}$. N = GWAS sample size; R^2^ is the proportion of variation in the exposure database explained by SNPs; β= effect estimate; EAF=effect allele frequency.

| **Supplementary Table S7.**  SNPs utilized as instruments for BP in final MR analysis on Prostate cancer. | | | | | | | | | | | | | | | | | |  |
| --- | --- | --- | --- | --- | --- | --- | --- | --- | --- | --- | --- | --- | --- | --- | --- | --- | --- | --- |
|  | BP | | | | | | | | | | | Prostate cancer | | | | | | |
|  | **SNP** | **CHR** | **POS** | **MAF** | **EA** | **OA** | **beta** | **eaf** | **pval** | **se** | **samplesize** | **beta** | **eaf** | **se** | **pval** | **samplesize** | **R^2^** | **F** |
| 1 | rs9996810 | 4 | 62468670 | 0.2406 | C | T | 0.5263 | 0.2406 | 4.52E-06 | 0.1148 | 218285 | 0.0019 | 0.2182 | 0.0009 | 0.0290 | 182625 | 9.63E-05 | 21.0174 |
| 2 | rs79334883 | 4 | 86211322 | 0.0421 | G | A | 1.2389 | 0.0421 | 3.38E-06 | 0.2667 | 218285 | 0.0010 | 0.0286 | 0.0022 | 0.6400 | 182625 | 9.88E-05 | 21.5786 |
| 3 | rs76965871 | 1 | 175729422 | 0.0111 | A | G | 3.0219 | 0.0111 | 6.37E-07 | 0.6069 | 218285 | 0.0036 | 0.0270 | 0.0022 | 0.1100 | 182625 | 1.14E-04 | 24.7926 |
| 4 | rs75308030 | 10 | 26019862 | 0.0063 | T | G | 4.7413 | 0.0063 | 6.23E-07 | 0.9513 | 218285 | -0.0027 | 0.0117 | 0.0036 | 0.4600 | 182625 | 1.14E-04 | 24.8402 |
| 5 | rs60559879 | 17 | 35192062 | 0.2705 | A | G | 0.5073 | 0.2705 | 3.99E-06 | 0.1100 | 218285 | 0.0001 | 0.2809 | 0.0008 | 0.9200 | 182625 | 9.74E-05 | 21.2687 |
| 6 | rs35604167 | 4 | 169382451 | 0.1114 | T | G | 0.7932 | 0.1114 | 1.09E-06 | 0.1627 | 218285 | -0.0001 | 0.1239 | 0.0011 | 0.9400 | 182625 | 1.09E-04 | 23.7677 |
| 7 | rs3094169 | 6 | 29829986 | 0.2806 | C | T | -0.5736 | 0.7194 | 3.04E-06 | 0.1229 | 218285 | 0.0006 | 0.8103 | 0.0009 | 0.5000 | 182625 | 9.98E-05 | 21.7826 |
| 8 | rs144298683 | 3 | 88344440 | 0.0139 | T | C | 2.7543 | 0.0139 | 1.05E-07 | 0.5178 | 218285 | 0.0004 | 0.0104 | 0.0036 | 0.9000 | 182625 | 1.30E-04 | 28.2940 |
| 9 | rs10483424 | 14 | 33591131 | 0.0089 | A | G | 3.3364 | 0.0089 | 5.73E-07 | 0.6673 | 218285 | -0.0111 | 0.0164 | 0.0028 | 0.0001 | 182625 | 1.15E-04 | 24.9983 |

Abbreviations: BP, Bullous pemphigoid; CHR, chromosome; MAF, minor allele frequency; MR, Mendelian randomization; POS, position; SNPs, single‐nucleotide polymorphisms.

Note(a): 14 independent genome wide significant SNPs were extracted from BP GWAS (P < 5E-06; LD R^2^ < 0.01, 1000kb); 12 of these instruments were found in Prostate cancer GWAS; 3 SNPs (rs145834662, rs147633772, rs11205458) were removed for being palindromic with intermediate allele frequencies (harmonization).

Note(b): At the more stringent threshold of P < 5E-08, No SNPs linked with other phenotypes were identified on the PhenoScancer website.

Note(c): $R^{2}=\frac{2\times\beta^{2}\times EAF\times(1-EAF)}{2\times\beta^{2}\times EAF\times\left( 1-EAF \right)+2\times{SE}^{2}\times N\times EAF\times(1-EAF)}$ ,$F=\frac{R^{2}\times(N-2)}{1-R^{2}}$. N = GWAS sample size; R^2^ is the proportion of variation in the exposure database explained by SNPs; β= effect estimate; EAF=effect allele frequency.

| **Supplementary Table S8.**  SNPs utilized as instruments for BP in final MR analysis on Breast cancer. | | | | | | | | | | | | | | | | | |  |
| --- | --- | --- | --- | --- | --- | --- | --- | --- | --- | --- | --- | --- | --- | --- | --- | --- | --- | --- |
|  | BP | | | | | | | | | | | Breast cancer | | | | | | |
|  | **SNP** | **CHR** | **POS** | **MAF** | **EA** | **OA** | **beta** | **eaf** | **pval** | **se** | **samplesize** | **beta** | **eaf** | **se** | **pval** | **samplesize** | **R^2^** | **F** |
| 1 | rs9996810 | 4 | 62468670 | 0.2406 | C | T | 0.5263 | 0.2406 | 4.52E-06 | 0.1148 | 218285 | 0.0002 | 0.2180 | 0.0009 | 0.8300 | 212402 | 9.6276E-05 | 21.0174 |
| 2 | rs79334883 | 4 | 86211322 | 0.0421 | G | A | 1.2389 | 0.0421 | 3.38E-06 | 0.2667 | 218285 | 0.0012 | 0.0288 | 0.0023 | 0.6000 | 212402 | 9.8846E-05 | 21.5786 |
| 3 | rs76965871 | 1 | 175729422 | 0.0111 | A | G | 3.0219 | 0.0111 | 6.37E-07 | 0.6069 | 218285 | -0.0005 | 0.0270 | 0.0023 | 0.8200 | 212402 | 1.1357E-04 | 24.7926 |
| 4 | rs75308030 | 10 | 26019862 | 0.0063 | T | G | 4.7413 | 0.0063 | 6.23E-07 | 0.9513 | 218285 | -0.0015 | 0.0114 | 0.0038 | 0.7000 | 212402 | 1.1379E-04 | 24.8402 |
| 5 | rs60559879 | 17 | 35192062 | 0.2705 | A | G | 0.5073 | 0.2705 | 3.99E-06 | 0.1100 | 218285 | 0.0002 | 0.2817 | 0.0008 | 0.8500 | 212402 | 9.7427E-05 | 21.2687 |
| 6 | rs35604167 | 4 | 169382451 | 0.1114 | T | G | 0.7932 | 0.1114 | 1.09E-06 | 0.1627 | 218285 | 0.0019 | 0.1244 | 0.0012 | 0.0960 | 212402 | 1.0887E-04 | 23.7677 |
| 7 | rs3094169 | 6 | 29829986 | 0.2806 | C | T | -0.5736 | 0.7194 | 3.04E-06 | 0.1229 | 218285 | 0.0028 | 0.8100 | 0.0010 | 0.0037 | 212402 | 9.9781E-05 | 21.7826 |
| 8 | rs144298683 | 3 | 88344440 | 0.0139 | T | C | 2.7543 | 0.0139 | 1.05E-07 | 0.5178 | 218285 | 0.0016 | 0.0106 | 0.0037 | 0.6800 | 212402 | 1.2960E-04 | 28.2940 |
| 9 | rs10483424 | 14 | 33591131 | 0.0089 | A | G | 3.3364 | 0.0089 | 5.73E-07 | 0.6673 | 218285 | -0.0019 | 0.0164 | 0.0030 | 0.5300 | 212402 | 1.1451E-04 | 24.9983 |

Abbreviations: BP, Bullous pemphigoid; CHR, chromosome; MAF, minor allele frequency; MR, Mendelian randomization; POS, position; SNPs, single‐nucleotide polymorphisms.

Note(a): 14 independent genome wide significant SNPs were extracted from BP GWAS (P < 5E-06; LD R^2^ < 0.01, 1000kb); 12 of these instruments were found in Breast cancer GWAS; 3 SNPs (rs145834662, rs147633772, rs11205458) were removed for being palindromic with intermediate allele frequencies (harmonization).

Note(b): At the more stringent threshold of P < 5E-08, No SNPs linked with other phenotypes were identified on the PhenoScancer website.

Note(c): $R^{2}=\frac{2\times\beta^{2}\times EAF\times(1-EAF)}{2\times\beta^{2}\times EAF\times\left( 1-EAF \right)+2\times{SE}^{2}\times N\times EAF\times(1-EAF)}$ ,$F=\frac{R^{2}\times(N-2)}{1-R^{2}}$. N = GWAS sample size; R^2^ is the proportion of variation in the exposure database explained by SNPs; β= effect estimate; EAF=effect allele frequency.

| **Supplementary Table S9.**  SNPs utilized as instruments for BP in final MR analysis on Ovarian cancer. | | | | | | | | | | | | | | | | | |  |
| --- | --- | --- | --- | --- | --- | --- | --- | --- | --- | --- | --- | --- | --- | --- | --- | --- | --- | --- |
|  | BP | | | | | | | | | | | Ovarian cancer | | | | | | |
|  | SNP | CHR | POS | MAF | EA | OA | beta | eaf | pval | se | samplesize | beta | eaf | se | pval | samplesize | R2 | F |
| 1 | rs9996810 | 4 | 62468670 | 0.2406 | C | T | 0.5263 | 0.2406 | 4.52E-06 | 0.1148 | 218285 | -0.0002 | 0.2179 | 0.0003 | 0.4600 | 199741 | 9.6276E-05 | 21.0174 |
| 2 | rs79334883 | 4 | 86211322 | 0.0421 | G | A | 1.2389 | 0.0421 | 3.38E-06 | 0.2667 | 218285 | 0.0010 | 0.0288 | 0.0007 | 0.1700 | 199741 | 9.8846E-05 | 21.5786 |
| 3 | rs76965871 | 1 | 175729422 | 0.0111 | A | G | 3.0219 | 0.0111 | 6.37E-07 | 0.6069 | 218285 | 0.0000 | 0.0270 | 0.0008 | 0.9700 | 199741 | 1.1357E-04 | 24.7926 |
| 4 | rs75308030 | 10 | 26019862 | 0.0063 | T | G | 4.7413 | 0.0063 | 6.23E-07 | 0.9513 | 218285 | 0.0011 | 0.0114 | 0.0012 | 0.4000 | 199741 | 1.1379E-04 | 24.8402 |
| 5 | rs60559879 | 17 | 35192062 | 0.2705 | A | G | 0.5073 | 0.2705 | 3.99E-06 | 0.1100 | 218285 | 0.0000 | 0.2817 | 0.0003 | 0.9100 | 199741 | 9.7427E-05 | 21.2687 |
| 6 | rs35604167 | 4 | 169382451 | 0.1114 | T | G | 0.7932 | 0.1114 | 1.09E-06 | 0.1627 | 218285 | 0.0003 | 0.1243 | 0.0004 | 0.4400 | 199741 | 1.0887E-04 | 23.7677 |
| 7 | rs3094169 | 6 | 29829986 | 0.2806 | C | T | -0.5736 | 0.7194 | 3.04E-06 | 0.1229 | 218285 | 0.0003 | 0.8096 | 0.0003 | 0.3200 | 199741 | 9.9781E-05 | 21.7826 |
| 8 | rs144298683 | 3 | 88344440 | 0.0139 | T | C | 2.7543 | 0.0139 | 1.05E-07 | 0.5178 | 218285 | -0.0002 | 0.0106 | 0.0012 | 0.8600 | 199741 | 1.2960E-04 | 28.2940 |
| 9 | rs10483424 | 14 | 33591131 | 0.0089 | A | G | 3.3364 | 0.0089 | 5.73E-07 | 0.6673 | 218285 | -0.0006 | 0.0164 | 0.0010 | 0.5300 | 199741 | 1.1451E-04 | 24.9983 |

Abbreviations: BP, Bullous pemphigoid; CHR, chromosome; MAF, minor allele frequency; MR, Mendelian randomization; POS, position; SNPs, single‐nucleotide polymorphisms.

Note(a): 14 independent genome wide significant SNPs were extracted from BP GWAS (P < 5E-06; LD R^2^ < 0.01, 1000kb); 12 of these instruments were found in Ovarian cancer GWAS; 3 SNPs (rs145834662, rs147633772, rs11205458) were removed for being palindromic with intermediate allele frequencies (harmonization).

Note(b): At the more stringent threshold of P < 5E-08, No SNPs linked with other phenotypes were identified on the PhenoScancer website.

Note(c): $R^{2}=\frac{2\times\beta^{2}\times EAF\times(1-EAF)}{2\times\beta^{2}\times EAF\times\left( 1-EAF \right)+2\times{SE}^{2}\times N\times EAF\times(1-EAF)}$ ,$F=\frac{R^{2}\times(N-2)}{1-R^{2}}$. N = GWAS sample size; R^2^ is the proportion of variation in the exposure database explained by SNPs; β= effect estimate; EAF=effect allele frequency.

| **Supplementary Table S10.**  SNPs utilized as instruments for BP in final MR analysis on Cervix cancer. | | | | | | | | | | | | | | | | | |  |
| --- | --- | --- | --- | --- | --- | --- | --- | --- | --- | --- | --- | --- | --- | --- | --- | --- | --- | --- |
|  | BP | | | | | | | | | | | Cervix cancer | | | | | | |
|  | SNP | CHR | POS | MAF | EA | OA | beta | eaf | pval | se | samplesize | beta | eaf | se | pval | samplesize | R2 | F |
| 1 | rs9996810 | 4 | 62468670 | 0.2406 | C | T | 0.5263 | 0.2406 | 4.52E-06 | 0.1148 | 218285 | -0.0001 | 0.2180 | 0.0002 | 0.7900 | 199086 | 9.6276E-05 | 21.0174 |
| 2 | rs79334883 | 4 | 86211322 | 0.0421 | G | A | 1.2389 | 0.0421 | 3.38E-06 | 0.2667 | 218285 | 0.0000 | 0.0288 | 0.0005 | 0.9500 | 199086 | 9.8846E-05 | 21.5786 |
| 3 | rs76965871 | 1 | 175729422 | 0.0111 | A | G | 3.0219 | 0.0111 | 6.37E-07 | 0.6069 | 218285 | -0.0005 | 0.0269 | 0.0005 | 0.3300 | 199086 | 1.1357E-04 | 24.7926 |
| 4 | rs60559879 | 17 | 35192062 | 0.2705 | A | G | 0.5073 | 0.2705 | 3.99E-06 | 0.1100 | 218285 | 0.0000 | 0.2817 | 0.0002 | 0.9300 | 199086 | 9.7427E-05 | 21.2687 |
| 5 | rs35604167 | 4 | 169382451 | 0.1114 | T | G | 0.7932 | 0.1114 | 1.09E-06 | 0.1627 | 218285 | -0.0002 | 0.1242 | 0.0003 | 0.4400 | 199086 | 1.0887E-04 | 23.7677 |
| 6 | rs3094169 | 6 | 29829986 | 0.2806 | C | T | -0.5736 | 0.7194 | 3.04E-06 | 0.1229 | 218285 | 0.0004 | 0.8096 | 0.0002 | 0.0780 | 199086 | 9.9781E-05 | 21.7826 |

Abbreviations: BP, Bullous pemphigoid; CHR, chromosome; MAF, minor allele frequency; MR, Mendelian randomization; POS, position; SNPs, single‐nucleotide polymorphisms.

Note(a): 14 independent genome wide significant SNPs were extracted from BP GWAS (P < 5E-06; LD R^2^ < 0.01, 1000kb); 8 of these instruments were found in Cervix cancer GWAS; 2 SNPs (rs147633772, rs11205458, rs145834662) were removed for being palindromic with intermediate allele frequencies (harmonization).

Note(b): At the more stringent threshold of P < 5E-08, No SNPs linked with other phenotypes were identified on the PhenoScancer website.

Note(c): $R^{2}=\frac{2\times\beta^{2}\times EAF\times(1-EAF)}{2\times\beta^{2}\times EAF\times\left( 1-EAF \right)+2\times{SE}^{2}\times N\times EAF\times(1-EAF)}$ ,$F=\frac{R^{2}\times(N-2)}{1-R^{2}}$. N = GWAS sample size; R^2^ is the proportion of variation in the exposure database explained by SNPs; β= effect estimate; EAF=effect allele frequency.

| **Supplementary Table S11.**  SNPs utilized as instruments for BP in final MR analysis on Leukaemia. | | | | | | | | | | | | | | | | | |  |
| --- | --- | --- | --- | --- | --- | --- | --- | --- | --- | --- | --- | --- | --- | --- | --- | --- | --- | --- |
|  | BP | | | | | | | | | | | Leukaemia | | | | | | |
|  | SNP | CHR | POS | MAF | EA | OA | beta | eaf | pval | se | samplesize | beta | eaf | se | pval | samplesize | R2 | F |
| 1 | rs9996810 | 4 | 62468670 | 0.2406 | C | T | 0.5263 | 0.2406 | 4.52E-06 | 0.1148 | 218285 | 0.0001 | 0.2179 | 0.0002 | 0.5000 | 373276 | 9.6276E-05 | 21.0174 |
| 2 | rs79334883 | 4 | 86211322 | 0.0421 | G | A | 1.2389 | 0.0421 | 3.38E-06 | 0.2667 | 218285 | -0.0001 | 0.0287 | 0.0004 | 0.8800 | 373276 | 9.8846E-05 | 21.5786 |
| 3 | rs76965871 | 1 | 175729422 | 0.0111 | A | G | 3.0219 | 0.0111 | 6.37E-07 | 0.6069 | 218285 | 0.0000 | 0.0269 | 0.0004 | 0.9200 | 373276 | 1.1357E-04 | 24.7926 |
| 4 | rs75308030 | 10 | 26019862 | 0.0063 | T | G | 4.7413 | 0.0063 | 6.23E-07 | 0.9513 | 218285 | 0.0001 | 0.0115 | 0.0007 | 0.8300 | 373276 | 1.1379E-04 | 24.8402 |
| 5 | rs60559879 | 17 | 35192062 | 0.2705 | A | G | 0.5073 | 0.2705 | 3.99E-06 | 0.1100 | 218285 | -0.0001 | 0.2813 | 0.0001 | 0.6000 | 373276 | 9.7427E-05 | 21.2687 |
| 6 | rs35604167 | 4 | 169382451 | 0.1114 | T | G | 0.7932 | 0.1114 | 1.09E-06 | 0.1627 | 218285 | -0.0004 | 0.1241 | 0.0002 | 0.0810 | 373276 | 1.0887E-04 | 23.7677 |
| 7 | rs3094169 | 6 | 29829986 | 0.2806 | C | T | -0.5736 | 0.7194 | 3.04E-06 | 0.1229 | 218285 | 0.0004 | 0.8099 | 0.0002 | 0.0310 | 373276 | 9.9781E-05 | 21.7826 |
| 8 | rs144298683 | 3 | 88344440 | 0.0139 | T | C | 2.7543 | 0.0139 | 1.05E-07 | 0.5178 | 218285 | 0.0011 | 0.0105 | 0.0007 | 0.1100 | 373276 | 1.2960E-04 | 28.2940 |
| 9 | rs10483424 | 14 | 33591131 | 0.0089 | A | G | 3.3364 | 0.0089 | 5.73E-07 | 0.6673 | 218285 | 0.0000 | 0.0165 | 0.0005 | 0.9700 | 373276 | 1.1451E-04 | 24.9983 |

Abbreviations: BP, Bullous pemphigoid; CHR, chromosome; MAF, minor allele frequency; MR, Mendelian randomization; POS, position; SNPs, single‐nucleotide polymorphisms.

Note(a): 14 independent genome wide significant SNPs were extracted from BP GWAS (P < 5E-06; LD R^2^ < 0.01, 1000kb); 12 of these instruments were found in Leukaemia GWAS; 3 SNPs (rs147633772, rs11205458, rs145834662) were removed for being palindromic with intermediate allele frequencies (harmonization).

Note(b): At the more stringent threshold of P < 5E-08, No SNPs linked with other phenotypes were identified on the PhenoScancer website.

Note(c): $R^{2}=\frac{2\times\beta^{2}\times EAF\times(1-EAF)}{2\times\beta^{2}\times EAF\times\left( 1-EAF \right)+2\times{SE}^{2}\times N\times EAF\times(1-EAF)}$ ,$F=\frac{R^{2}\times(N-2)}{1-R^{2}}$. N = GWAS sample size; R^2^ is the proportion of variation in the exposure database explained by SNPs; β= effect estimate; EAF=effect allele frequency.

| **Supplementary Table S12.**  SNPs utilized as instruments for BP in final MR analysis on Lymphomas. | | | | | | | | | | | | | | | | | |  |
| --- | --- | --- | --- | --- | --- | --- | --- | --- | --- | --- | --- | --- | --- | --- | --- | --- | --- | --- |
|  | BP | | | | | | | | | | | Lymphomas | | | | | | |
|  | SNP | CHR | POS | MAF | EA | OA | beta | eaf | pval | se | samplesize | beta | eaf | se | pval | samplesize | R2 | F |
| 1 | rs9996810 | 4 | 62468670 | 0.2406 | C | T | 0.5263 | 0.2406 | 4.52E-06 | 0.1148 | 218285 | 0.0003 | 0.2174 | 0.0002 | 0.1586 | 361194 | 9.6276E-05 | 21.0174 |
| 2 | rs79334883 | 4 | 86211322 | 0.0421 | G | A | 1.2389 | 0.0421 | 3.38E-06 | 0.2667 | 218285 | -0.0001 | 0.0285 | 0.0005 | 0.8107 | 361194 | 9.8846E-05 | 21.5786 |
| 3 | rs76965871 | 1 | 175729422 | 0.0111 | A | G | 3.0219 | 0.0111 | 6.37E-07 | 0.6069 | 218285 | -0.0008 | 0.0277 | 0.0005 | 0.1143 | 361194 | 1.1357E-04 | 24.7926 |
| 4 | rs75308030 | 10 | 26019862 | 0.0063 | T | G | 4.7413 | 0.0063 | 6.23E-07 | 0.9513 | 218285 | 0.0008 | 0.0117 | 0.0008 | 0.3530 | 361194 | 1.1379E-04 | 24.8402 |
| 5 | rs60559879 | 17 | 35192062 | 0.2705 | A | G | 0.5073 | 0.2705 | 3.99E-06 | 0.1100 | 218285 | -0.0001 | 0.2814 | 0.0002 | 0.5406 | 361194 | 9.7427E-05 | 21.2687 |
| 6 | rs35604167 | 4 | 169382451 | 0.1114 | T | G | 0.7932 | 0.1114 | 1.09E-06 | 0.1627 | 218285 | 0.0000 | 0.1239 | 0.0003 | 0.9256 | 361194 | 1.0887E-04 | 23.7677 |
| 7 | rs3094169 | 6 | 29829986 | 0.2806 | C | T | -0.5736 | 0.7194 | 3.04E-06 | 0.1229 | 218285 | 0.0000 | 0.8115 | 0.0002 | 0.9134 | 361194 | 9.9781E-05 | 21.7826 |
| 8 | rs200148 | 6 | 143345696 | 0.4040 | A | G | -0.479 | 0.5960 | 1.27E-06 | 0.0989 | 218285 | -0.0003 | 0.6014 | 0.0002 | 0.0753 | 361194 | 1.0745E-04 | 23.4571 |
| 9 | rs144298683 | 3 | 88344440 | 0.0139 | T | C | 2.7543 | 0.0139 | 1.05E-07 | 0.5178 | 218285 | 0.0011 | 0.0105 | 0.0008 | 0.1856 | 361194 | 1.2960E-04 | 28.2940 |
| 10 | rs10483424 | 14 | 33591131 | 0.0089 | A | G | 3.3364 | 0.0089 | 5.73E-07 | 0.6673 | 218285 | -0.0002 | 0.0166 | 0.0006 | 0.7082 | 361194 | 1.1451E-04 | 24.9983 |

Abbreviations: BP, Bullous pemphigoid; CHR, chromosome; MAF, minor allele frequency; MR, Mendelian randomization; POS, position; SNPs, single‐nucleotide polymorphisms.

Note(a): 14 independent genome wide significant SNPs were extracted from BP GWAS (P < 5E-06; LD R^2^ < 0.01, 1000kb); 14 of these instruments were found in Lymphomas GWAS; 4 SNPs (rs147633772, rs11205458, rs145834662, rs6550374) were removed for being palindromic with intermediate allele frequencies (harmonization).

Note(b): At the more stringent threshold of P < 5E-08, No SNPs linked with other phenotypes were identified on the PhenoScancer website.

Note(c): $R^{2}=\frac{2\times\beta^{2}\times EAF\times(1-EAF)}{2\times\beta^{2}\times EAF\times\left( 1-EAF \right)+2\times{SE}^{2}\times N\times EAF\times(1-EAF)}$ ,$F=\frac{R^{2}\times(N-2)}{1-R^{2}}$. N = GWAS sample size; R^2^ is the proportion of variation in the exposure database explained by SNPs; β= effect estimate; EAF=effect allele frequency.

| **Supplementary Table S13.**  SNPs utilized as instruments for BP in final MR analysis on Oesophageal cancer. | | | | | | | | | | | | | | | | | |  |
| --- | --- | --- | --- | --- | --- | --- | --- | --- | --- | --- | --- | --- | --- | --- | --- | --- | --- | --- |
|  | BP | | | | | | | | | | | Oesophageal cancer | | | | | | |
|  | SNP | CHR | POS | MAF | EA | OA | beta | eaf | pval | se | samplesize | beta | eaf | se | pval | samplesize | R2 | F |
| 1 | rs9996810 | 4 | 62468670 | 0.2406 | C | T | 0.5263 | 0.2406 | 4.52E-06 | 0.1148 | 218285 | -0.0001 | 0.2179 | 0.0001 | 0.3400 | 372756 | 9.6276E-05 | 21.0174 |
| 2 | rs79334883 | 4 | 86211322 | 0.0421 | G | A | 1.2389 | 0.0421 | 3.38E-06 | 0.2667 | 218285 | 0.0004 | 0.0287 | 0.0003 | 0.2200 | 372756 | 9.8846E-05 | 21.5786 |
| 3 | rs76965871 | 1 | 175729422 | 0.0111 | A | G | 3.0219 | 0.0111 | 6.37E-07 | 0.6069 | 218285 | -0.0002 | 0.0269 | 0.0003 | 0.5200 | 372756 | 1.1357E-04 | 24.7926 |
| 4 | rs60559879 | 17 | 35192062 | 0.2705 | A | G | 0.5073 | 0.2705 | 3.99E-06 | 0.1100 | 218285 | 0.0001 | 0.2814 | 0.0001 | 0.5800 | 372756 | 9.7427E-05 | 21.2687 |
| 5 | rs35604167 | 4 | 169382451 | 0.1114 | T | G | 0.7932 | 0.1114 | 1.09E-06 | 0.1627 | 218285 | 0.0000 | 0.1241 | 0.0002 | 0.7600 | 372756 | 1.0887E-04 | 23.7677 |
| 6 | rs3094169 | 6 | 29829986 | 0.2806 | C | T | -0.5736 | 0.7194 | 3.04E-06 | 0.1229 | 218285 | 0.0000 | 0.8099 | 0.0001 | 0.7200 | 372756 | 9.9781E-05 | 21.7826 |

Abbreviations: BP, Bullous pemphigoid; CHR, chromosome; MAF, minor allele frequency; MR, Mendelian randomization; POS, position; SNPs, single‐nucleotide polymorphisms.

Note(a): 14 independent genome wide significant SNPs were extracted from BP GWAS (P < 5E-06; LD R^2^ < 0.01, 1000kb); 9 of these instruments were found in Oesophageal cancer GWAS; 3 SNPs (rs11205458, rs145834662, rs147633772) were removed for being palindromic with intermediate allele frequencies (harmonization).

Note(b): At the more stringent threshold of P < 5E-08, No SNPs linked with other phenotypes were identified on the PhenoScancer website.

Note(c): $R^{2}=\frac{2\times\beta^{2}\times EAF\times(1-EAF)}{2\times\beta^{2}\times EAF\times\left( 1-EAF \right)+2\times{SE}^{2}\times N\times EAF\times(1-EAF)}$ ,$F=\frac{R^{2}\times(N-2)}{1-R^{2}}$. N = GWAS sample size; R^2^ is the proportion of variation in the exposure database explained by SNPs; β= effect estimate; EAF=effect allele frequency.

| **Supplementary Table S14.**  SNPs utilized as instruments for BP in final MR analysis on Liver cell carcinoma. | | | | | | | | | | | | | | | | | |  |
| --- | --- | --- | --- | --- | --- | --- | --- | --- | --- | --- | --- | --- | --- | --- | --- | --- | --- | --- |
|  | BP | | | | | | | | | | | Liver cell carcinoma | | | | | | |
|  | SNP | CHR | POS | MAF | EA | OA | beta | eaf | pval | se | samplesize | beta | eaf | se | pval | samplesize | R2 | F |
| 1 | rs9996810 | 4 | 62468670 | 0.2406 | C | T | 0.5263 | 0.2406 | 4.52E-06 | 0.1148 | 218285 | -0.0001 | 0.2179 | 0.0001 | 0.3700 | 372184 | 9.6276E-05 | 21.0174 |
| 2 | rs60559879 | 17 | 35192062 | 0.2705 | A | G | 0.5073 | 0.2705 | 3.99E-06 | 0.1100 | 218285 | 0.0000 | 0.2814 | 0.0001 | 0.7500 | 372184 | 9.7427E-05 | 21.2687 |
| 3 | rs35604167 | 4 | 169382451 | 0.1114 | T | G | 0.7932 | 0.1114 | 1.09E-06 | 0.1627 | 218285 | 0.0000 | 0.1241 | 0.0001 | 0.6900 | 372184 | 1.0887E-04 | 23.7677 |
| 4 | rs3094169 | 6 | 29829986 | 0.2806 | C | T | -0.5736 | 0.7194 | 3.04E-06 | 0.1229 | 218285 | -0.0001 | 0.8098 | 0.0001 | 0.3900 | 372184 | 9.9781E-05 | 21.7826 |

Abbreviations: BP, Bullous pemphigoid; CHR, chromosome; MAF, minor allele frequency; MR, Mendelian randomization; POS, position; SNPs, single‐nucleotide polymorphisms.

Note(a): 14 independent genome wide significant SNPs were extracted from BP GWAS (P < 5E-06; LD R^2^ < 0.01, 1000kb); 6 of these instruments were found in Liver cell carcinoma GWAS; 2 SNPs (rs11205458, rs147633772) were removed for being palindromic with intermediate allele frequencies (harmonization).

Note(b): At the more stringent threshold of P < 5E-08, No SNPs linked with other phenotypes were identified on the PhenoScancer website.

Note(c): $R^{2}=\frac{2\times\beta^{2}\times EAF\times(1-EAF)}{2\times\beta^{2}\times EAF\times\left( 1-EAF \right)+2\times{SE}^{2}\times N\times EAF\times(1-EAF)}$ ,$F=\frac{R^{2}\times(N-2)}{1-R^{2}}$. N = GWAS sample size; R^2^ is the proportion of variation in the exposure database explained by SNPs; β= effect estimate; EAF=effect allele frequency.

| **Supplementary Table S15.** Five Mendelian randomization approaches were employed to investigate the association between BP and malignant neoplasms. | | | | | | | | |
| --- | --- | --- | --- | --- | --- | --- | --- | --- |
| Outcome | Method | nSNP | b | se | pval | or | or_lci95 | or_uci95 |
| Melanoma skin cancer | MR Egger | 9 | -0.0002 | 0.0002 | 0.2120 | 0.9998 | 0.9994 | 1.0001 |
| Melanoma skin cancer | Weighted median | 9 | -0.0001 | 0.0002 | 0.5869 | 0.9999 | 0.9996 | 1.0002 |
| Melanoma skin cancer | Inverse variance weighted | 9 | -0.0001 | 0.0001 | 0.2768 | 0.9999 | 0.9996 | 1.0001 |
| Melanoma skin cancer | Simple mode | 9 | 0.0001 | 0.0003 | 0.8396 | 1.0001 | 0.9995 | 1.0006 |
| Melanoma skin cancer | Weighted mode | 9 | 0.0001 | 0.0002 | 0.7548 | 1.0001 | 0.9996 | 1.0005 |
| Malignant non-melanoma skin cancer | MR Egger | 9 | -0.0007 | 0.0005 | 0.1602 | 0.9993 | 0.9984 | 1.0002 |
| Malignant non-melanoma skin cancer | Weighted median | 9 | -0.0007 | 0.0004 | 0.0643 | 0.9993 | 0.9985 | 1.0000 |
| Malignant non-melanoma skin cancer | Inverse variance weighted | 9 | -0.0005 | 0.0003 | 0.0864 | 0.9995 | 0.9989 | 1.0001 |
| Malignant non-melanoma skin cancer | Simple mode | 9 | -0.0012 | 0.0007 | 0.1151 | 0.9988 | 0.9974 | 1.0001 |
| Malignant non-melanoma skin cancer | Weighted mode | 9 | -0.0011 | 0.0005 | 0.0753 | 0.9989 | 0.9979 | 1.0000 |
| Lung cancer | MR Egger | 9 | 0.0003 | 0.0002 | 0.1747 | 1.0003 | 0.9999 | 1.0008 |
| Lung cancer | Weighted median | 9 | 0.0003 | 0.0001 | 0.0624 | 1.0003 | 1.0000 | 1.0006 |
| Lung cancer | Inverse variance weighted | 9 | 0.0001 | 0.0002 | 0.5787 | 1.0001 | 0.9998 | 1.0004 |
| Lung cancer | Simple mode | 9 | 0.0004 | 0.0003 | 0.2120 | 1.0004 | 0.9998 | 1.0011 |
| Lung cancer | Weighted mode | 9 | 0.0003 | 0.0002 | 0.0630 | 1.0003 | 1.0000 | 1.0006 |
| Bladder cancer | MR Egger | 9 | -0.0001 | 0.0001 | 0.3767 | 0.9999 | 0.9997 | 1.0001 |
| Bladder cancer | Weighted median | 9 | -0.0001 | 0.0001 | 0.3124 | 0.9999 | 0.9997 | 1.0001 |
| Bladder cancer | Inverse variance weighted | 9 | 0.0000 | 0.0001 | 0.8029 | 1.0000 | 0.9998 | 1.0001 |
| Bladder cancer | Simple mode | 9 | 0.0000 | 0.0001 | 0.7202 | 1.0000 | 0.9997 | 1.0002 |
| Bladder cancer | Weighted mode | 9 | -0.0001 | 0.0001 | 0.4765 | 0.9999 | 0.9997 | 1.0001 |
| Colorectal cancer | MR Egger | 9 | 0.0004 | 0.0003 | 0.2187 | 1.0004 | 0.9998 | 1.0009 |
| Colorectal cancer | Weighted median | 9 | 0.0002 | 0.0002 | 0.2518 | 1.0002 | 0.9998 | 1.0006 |
| Colorectal cancer | Inverse variance weighted | 9 | 0.0002 | 0.0002 | 0.3042 | 1.0002 | 0.9998 | 1.0005 |
| Colorectal cancer | Simple mode | 9 | 0.0000 | 0.0003 | 0.9185 | 1.0000 | 0.9993 | 1.0006 |
| Colorectal cancer | Weighted mode | 9 | 0.0003 | 0.0002 | 0.2649 | 1.0003 | 0.9998 | 1.0007 |
| Prostate cancer | MR Egger | 9 | -0.0009 | 0.0009 | 0.3736 | 0.9991 | 0.9974 | 1.0009 |
| Prostate cancer | Weighted median | 9 | 0.0000 | 0.0006 | 0.9882 | 1.0000 | 0.9989 | 1.0011 |
| Prostate cancer | Inverse variance weighted | 9 | -0.0003 | 0.0006 | 0.6542 | 0.9997 | 0.9985 | 1.0009 |
| Prostate cancer | Simple mode | 9 | 0.0001 | 0.0009 | 0.9315 | 1.0001 | 0.9984 | 1.0018 |
| Prostate cancer | Weighted mode | 9 | -0.0002 | 0.0008 | 0.7859 | 0.9998 | 0.9983 | 1.0013 |
| Breast cancer | MR Egger | 9 | -0.0001 | 0.0007 | 0.9193 | 0.9999 | 0.9985 | 1.0013 |
| Breast cancer | Weighted median | 9 | -0.0002 | 0.0005 | 0.6322 | 0.9998 | 0.9989 | 1.0007 |
| Breast cancer | Inverse variance weighted | 9 | -0.0002 | 0.0005 | 0.6981 | 0.9998 | 0.9989 | 1.0007 |
| Breast cancer | Simple mode | 9 | 0.0002 | 0.0007 | 0.8025 | 1.0002 | 0.9989 | 1.0014 |
| Breast cancer | Weighted mode | 9 | -0.0002 | 0.0005 | 0.6617 | 0.9998 | 0.9988 | 1.0007 |
| Ovarian cancer | MR Egger | 9 | 0.0001 | 0.0002 | 0.5679 | 1.0001 | 0.9998 | 1.0005 |
| Ovarian cancer | Weighted median | 9 | 0.0000 | 0.0002 | 0.9603 | 1.0000 | 0.9997 | 1.0003 |
| Ovarian cancer | Inverse variance weighted | 9 | 0.0000 | 0.0001 | 0.8566 | 1.0000 | 0.9998 | 1.0003 |
| Ovarian cancer | Simple mode | 9 | -0.0001 | 0.0002 | 0.7932 | 0.9999 | 0.9995 | 1.0004 |
| Ovarian cancer | Weighted mode | 9 | 0.0000 | 0.0002 | 0.9595 | 1.0000 | 0.9996 | 1.0004 |
| cervical cancer | MR Egger | 6 | -0.0001 | 0.0002 | 0.5223 | 0.9999 | 0.9994 | 1.0003 |
| cervical cancer | Weighted median | 6 | -0.0002 | 0.0002 | 0.2866 | 0.9998 | 0.9995 | 1.0001 |
| cervical cancer | Inverse variance weighted | 6 | -0.0002 | 0.0001 | 0.1004 | 0.9998 | 0.9996 | 1.0000 |
| cervical cancer | Simple mode | 6 | -0.0001 | 0.0002 | 0.6405 | 0.9999 | 0.9995 | 1.0003 |
| cervical cancer | Weighted mode | 6 | -0.0002 | 0.0002 | 0.3798 | 0.9998 | 0.9995 | 1.0002 |
| Leukaemia | MR Egger | 9 | 0.0001 | 0.0001 | 0.4458 | 1.0001 | 0.9999 | 1.0003 |
| Leukaemia | Weighted median | 9 | 0.0000 | 0.0001 | 0.9881 | 1.0000 | 0.9998 | 1.0002 |
| Leukaemia | Inverse variance weighted | 9 | 0.0000 | 0.0001 | 0.7075 | 1.0000 | 0.9998 | 1.0001 |
| Leukaemia | Simple mode | 9 | 0.0000 | 0.0001 | 0.8889 | 1.0000 | 0.9997 | 1.0002 |
| Leukaemia | Weighted mode | 9 | 0.0000 | 0.0001 | 0.9782 | 1.0000 | 0.9998 | 1.0002 |
| Lymphomas | MR Egger | 10 | -0.0001 | 0.0001 | 0.6103 | 0.9999 | 0.9997 | 1.0002 |
| Lymphomas | Weighted median | 10 | 0.0000 | 0.0001 | 0.9728 | 1.0000 | 0.9998 | 1.0002 |
| Lymphomas | Inverse variance weighted | 10 | 0.0000 | 0.0001 | 0.6856 | 1.0000 | 0.9999 | 1.0002 |
| Lymphomas | Simple mode | 10 | -0.0001 | 0.0002 | 0.8061 | 0.9999 | 0.9996 | 1.0003 |
| Lymphomas | Weighted mode | 10 | -0.0001 | 0.0002 | 0.7022 | 0.9999 | 0.9996 | 1.0002 |
| Oesophageal cancer | MR Egger | 6 | 0.0000 | 0.0001 | 0.8438 | 1.0000 | 0.9997 | 1.0002 |
| Oesophageal cancer | Weighted median | 6 | 0.0000 | 0.0001 | 0.6586 | 1.0000 | 0.9998 | 1.0001 |
| Oesophageal cancer | Inverse variance weighted | 6 | 0.0000 | 0.0001 | 0.8331 | 1.0000 | 0.9998 | 1.0001 |
| Oesophageal cancer | Simple mode | 6 | 0.0000 | 0.0001 | 0.7988 | 1.0000 | 0.9997 | 1.0002 |
| Oesophageal cancer | Weighted mode | 6 | -0.0001 | 0.0001 | 0.5904 | 0.9999 | 0.9997 | 1.0001 |
| Liver cell carcinoma | MR Egger | 4 | 0.0001 | 0.0003 | 0.6991 | 1.0001 | 0.9995 | 1.0007 |
| Liver cell carcinoma | Weighted median | 4 | 0.0000 | 0.0001 | 0.5682 | 1.0000 | 0.9999 | 1.0002 |
| Liver cell carcinoma | Inverse variance weighted | 4 | 0.0000 | 0.0001 | 0.7070 | 1.0000 | 0.9999 | 1.0001 |
| Liver cell carcinoma | Simple mode | 4 | 0.0000 | 0.0001 | 0.6756 | 1.0000 | 0.9999 | 1.0002 |
| Liver cell carcinoma | Weighted mode | 4 | 0.0000 | 0.0001 | 0.6646 | 1.0000 | 0.9999 | 1.0002 |

Abbreviations: BP, Bullous pemphigoid; MR, Mendelian randomization; nSNP, number of single‐nucleotide polymorphism.

| **Supplementary Table S16.** Analysis of heterogeneity. | | | | |
| --- | --- | --- | --- | --- |
| Outcome | Method | Q | P for Q | P for random-effects IVW |
| Melanoma skin cancer | MR Egger | 3.8005 | 0.8025 |  |
| Melanoma skin cancer | Inverse variance weighted | 4.5400 | 0.8054 |  |
| Malignant non-melanoma skin cancer | MR Egger | 9.9890 | 0.1892 |  |
| Malignant non-melanoma skin cancer | Inverse variance weighted | 10.5025 | 0.2315 |  |
| Lung cancer | MR Egger | 17.3410 | 0.0153 |  |
| Lung cancer | Inverse variance weighted | 23.0512 | 0.0033 | 0.5787 |
| Bladder cancer | MR Egger | 8.2923 | 0.3075 |  |
| Bladder cancer | Inverse variance weighted | 9.6388 | 0.2913 |  |
| Colorectal cancer | MR Egger | 12.0574 | 0.0987 |  |
| Colorectal cancer | Inverse variance weighted | 13.4482 | 0.0973 |  |
| Prostate cancer | MR Egger | 20.7916 | 0.0041 |  |
| Prostate cancer | Inverse variance weighted | 23.1602 | 0.0032 | 0.6542 |
| Breast cancer | MR Egger | 12.0214 | 0.0999 |  |
| Breast cancer | Inverse variance weighted | 12.0920 | 0.1471 |  |
| Ovarian cancer | MR Egger | 4.7094 | 0.6954 |  |
| Ovarian cancer | Inverse variance weighted | 5.1405 | 0.7425 |  |
| cervical cancer | MR Egger | 1.9376 | 0.7472 |  |
| cervical cancer | Inverse variance weighted | 2.0278 | 0.8453 |  |
| Leukaemia | MR Egger | 8.2022 | 0.3151 |  |
| Leukaemia | Inverse variance weighted | 10.8606 | 0.2097 |  |
| Lymphomas | MR Egger | 9.1994 | 0.3258 |  |
| Lymphomas | Inverse variance weighted | 10.6586 | 0.2998 |  |
| Oesophageal cancer | MR Egger | 3.2994 | 0.5090 |  |
| Oesophageal cancer | Inverse variance weighted | 3.3112 | 0.6521 |  |
| Liver cell carcinoma | MR Egger | 1.5215 | 0.4673 |  |
| Liver cell carcinoma | Inverse variance weighted | 1.6716 | 0.6433 |  |

Abbreviations: IVW, inverse variance weighted; MR, Mendelian randomization.

| **Supplementary Table S17.** Analysis of horizontal pleiotropy. | | | |
| --- | --- | --- | --- |
| Outcome | MR-Egger test | se | pval |
| Melanoma skin cancer | 0.0002 | 0.0002 | 0.4183 |
| Malignant non-melanoma skin cancer | 0.0003 | 0.0005 | 0.5675 |
| Lung cancer | -0.0004 | 0.0003 | 0.1727 |
| Bladder cancer | 0.0001 | 0.0001 | 0.3217 |
| Colorectal cancer | -0.0003 | 0.0003 | 0.3987 |
| Prostate cancer | 0.0009 | 0.0011 | 0.4015 |
| Breast cancer | -0.0002 | 0.0008 | 0.8451 |
| Ovarian cancer | -0.0001 | 0.0002 | 0.5324 |
| cervical cancer | -0.0001 | 0.0002 | 0.7789 |
| Leukaemia | -0.0002 | 0.0001 | 0.1757 |
| Lymphomas | 0.0001 | 0.0001 | 0.2926 |
| Oesophageal cancer | 0.0000 | 0.0001 | 0.9187 |
| Liver cell carcinoma | -0.0001 | 0.0002 | 0.7358 |
| Abbreviations: MR, Mendelian randomization. | | | |


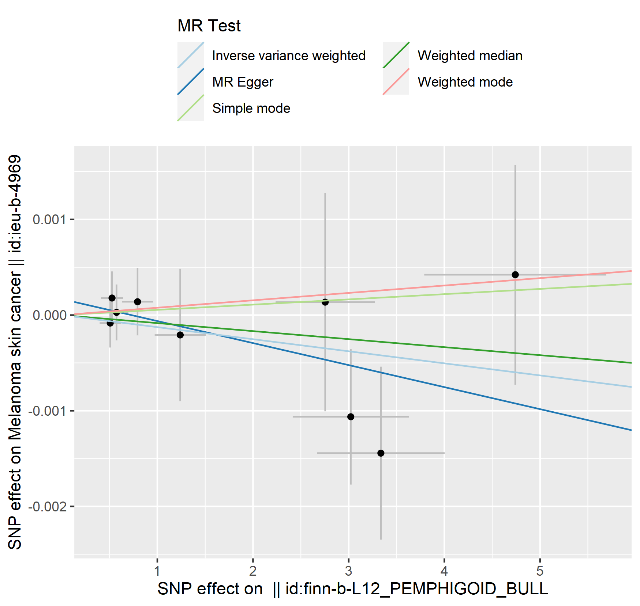

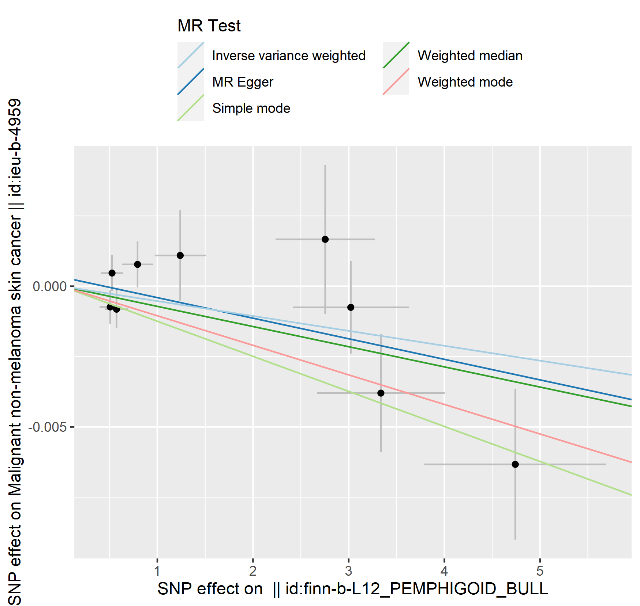

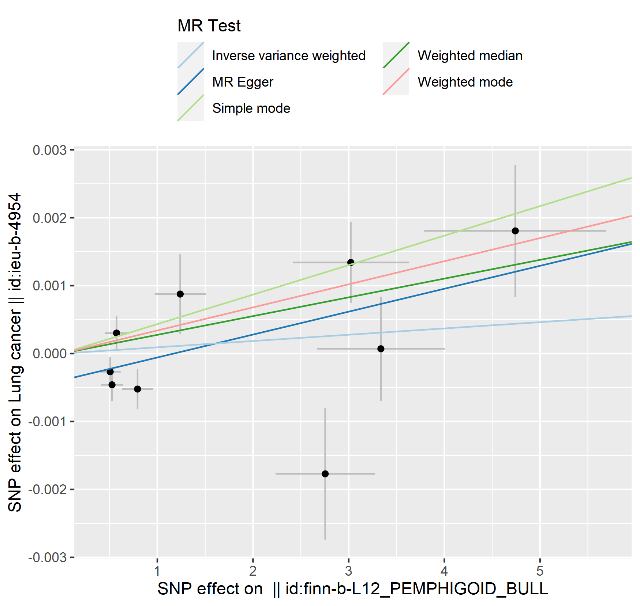


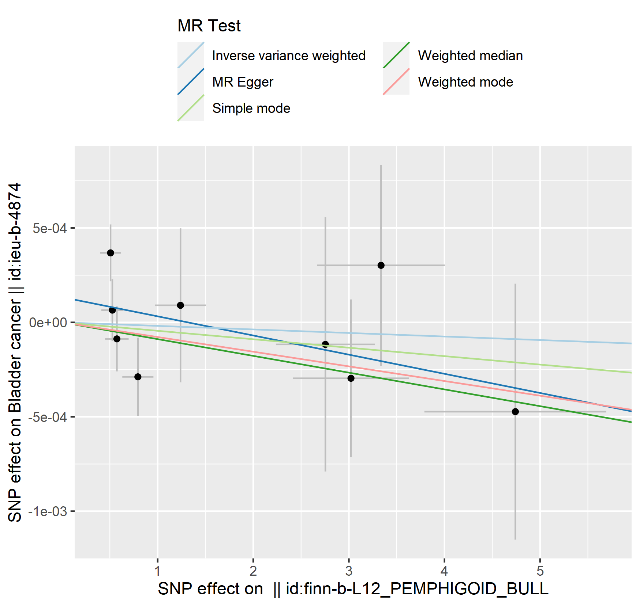

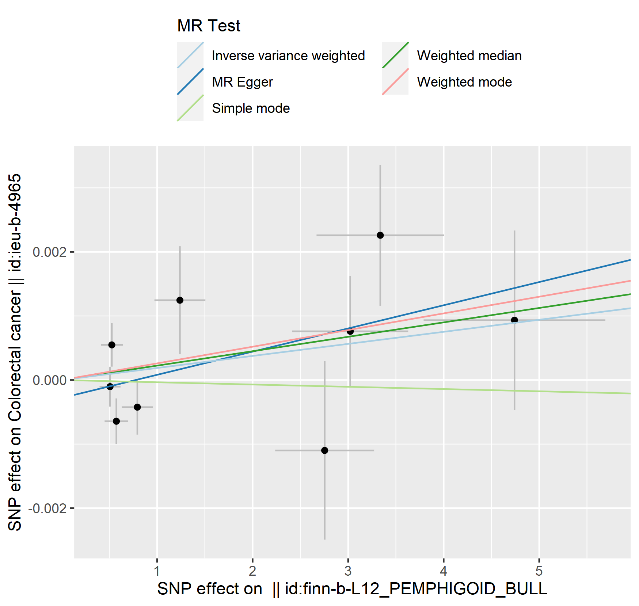

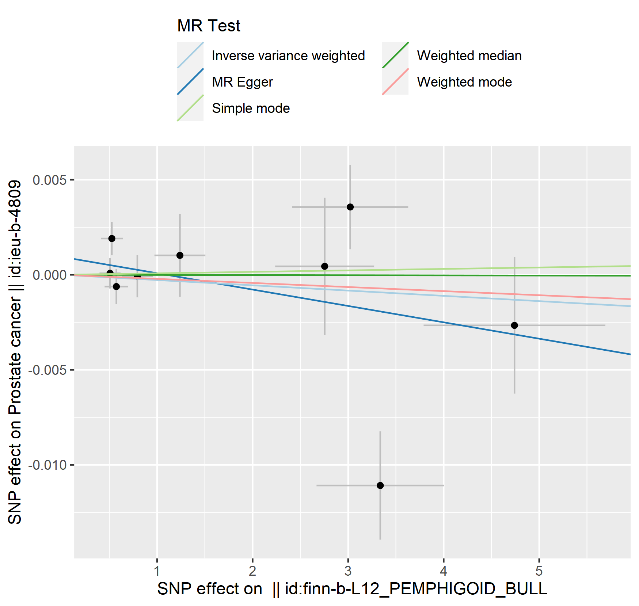


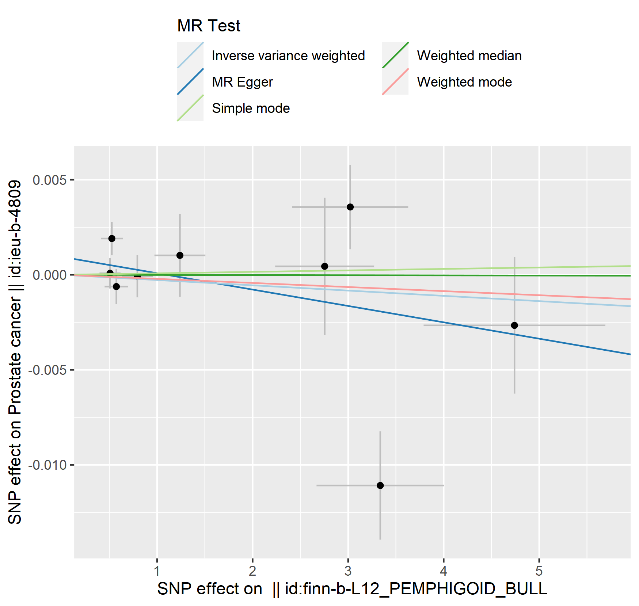

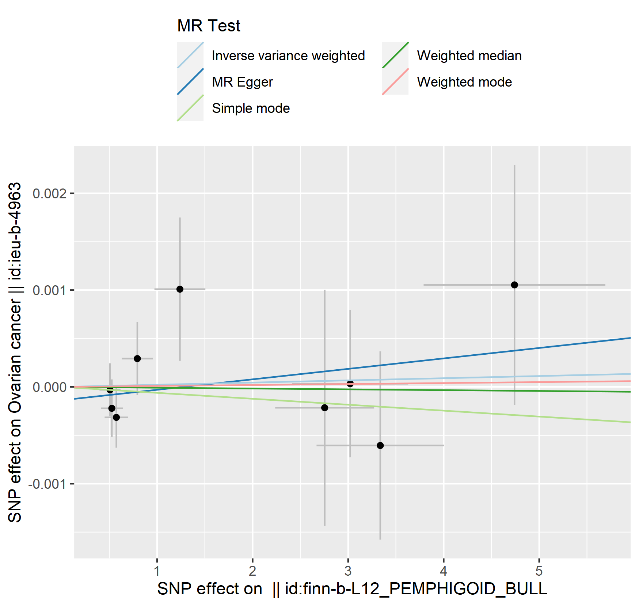

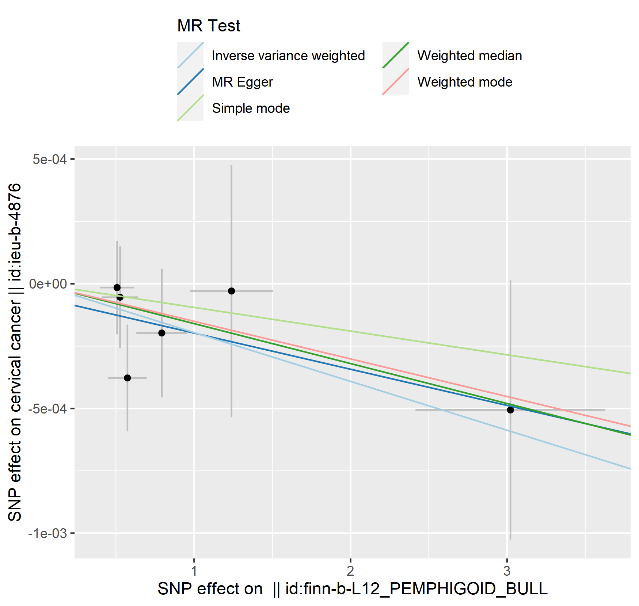


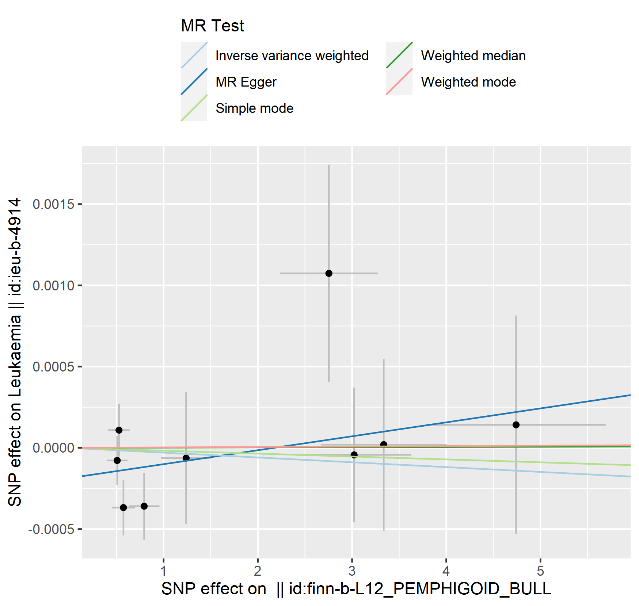

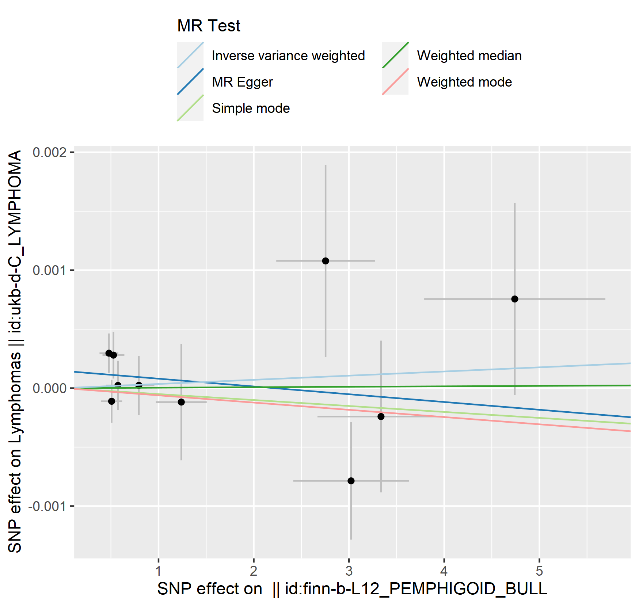

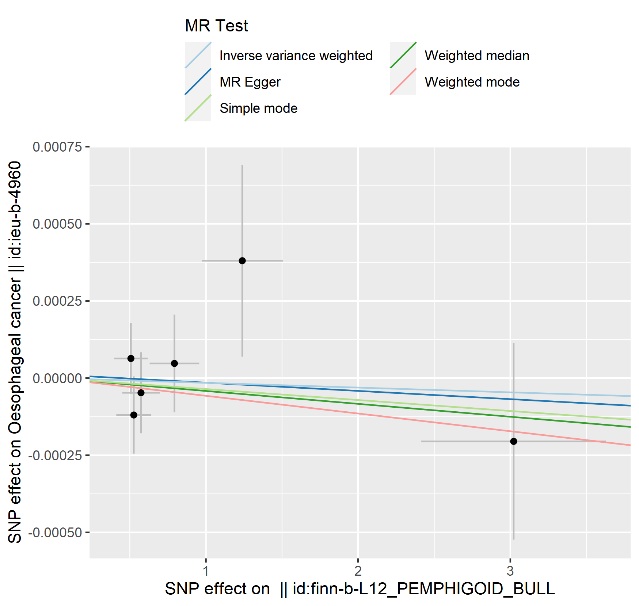


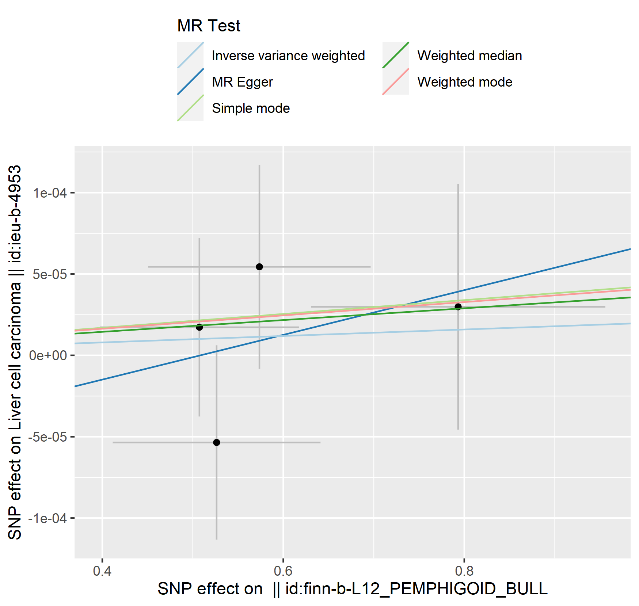


**Supplementary Figure 1** The scatter plots of the association between BP and 13 types of malignant tumors in MR analysis.


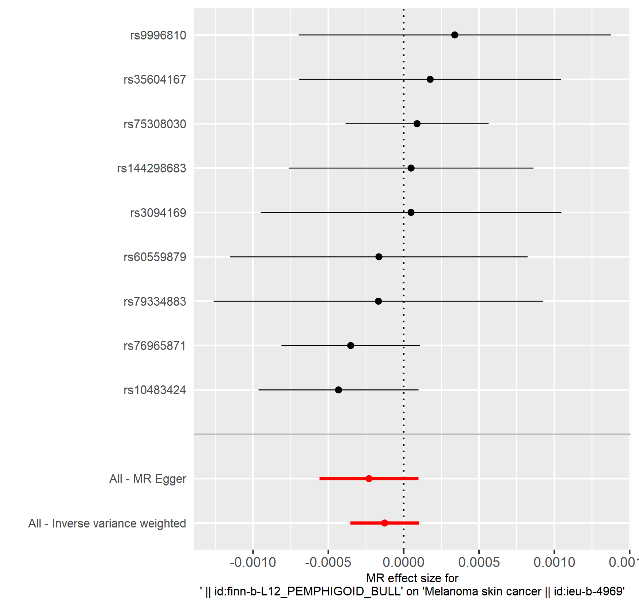

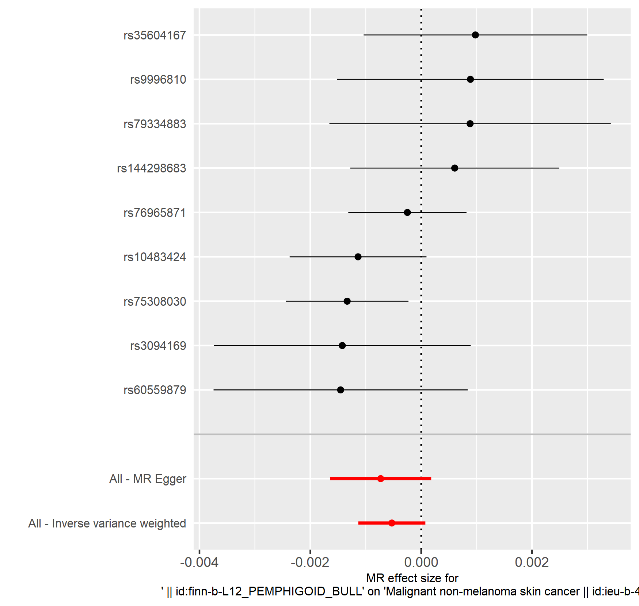

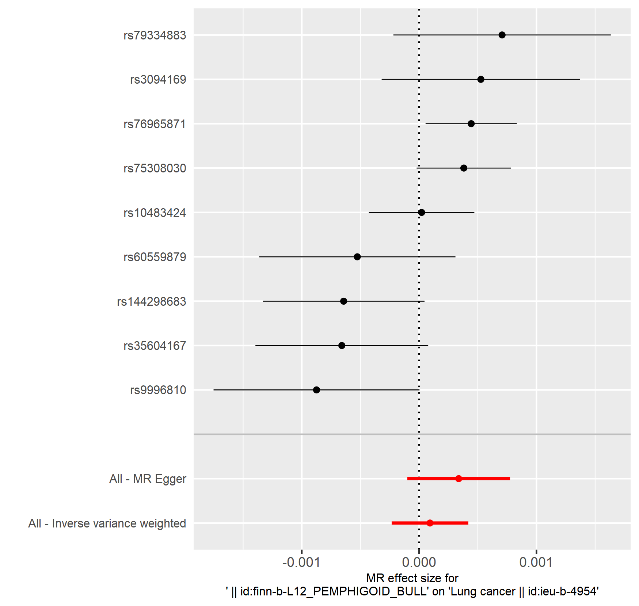


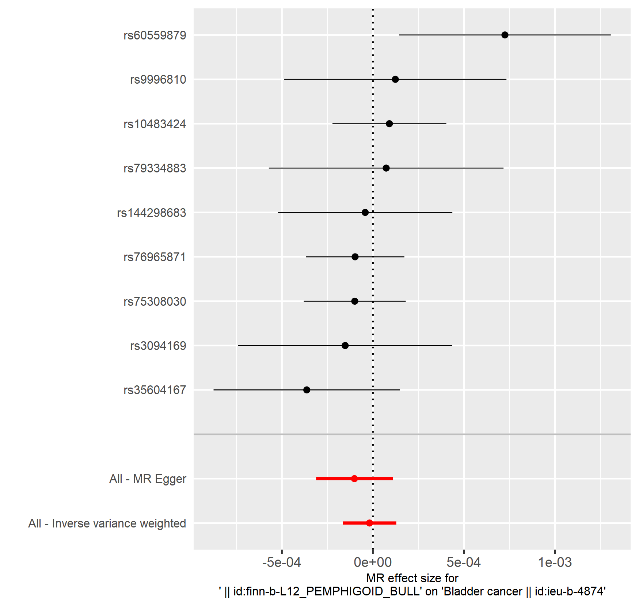

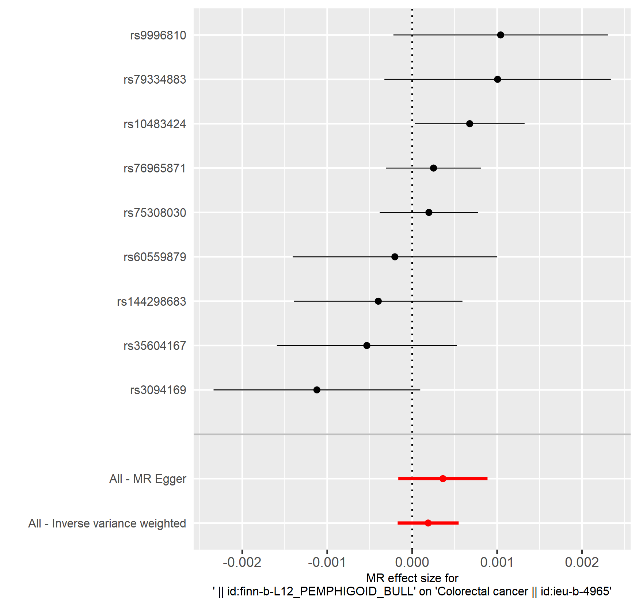

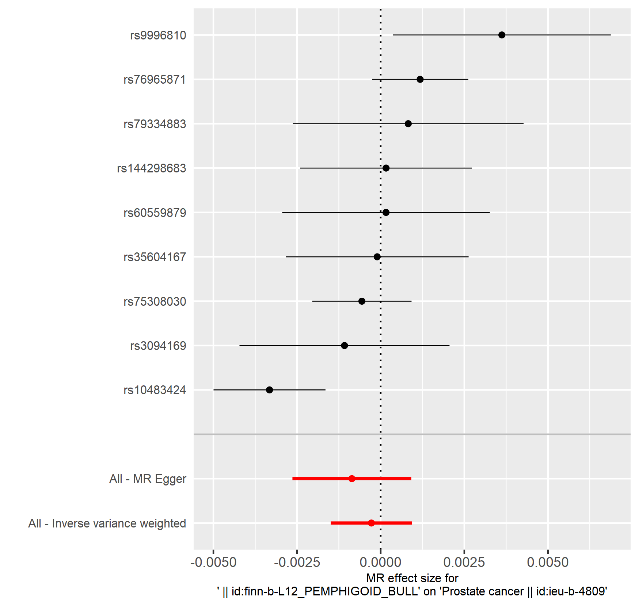


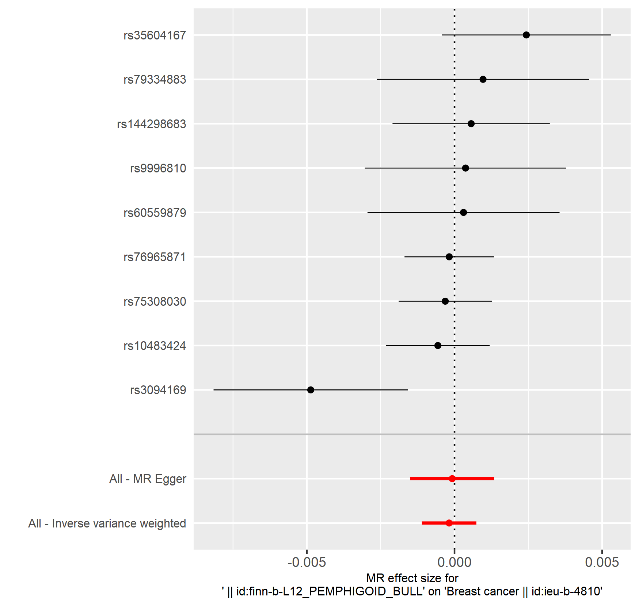

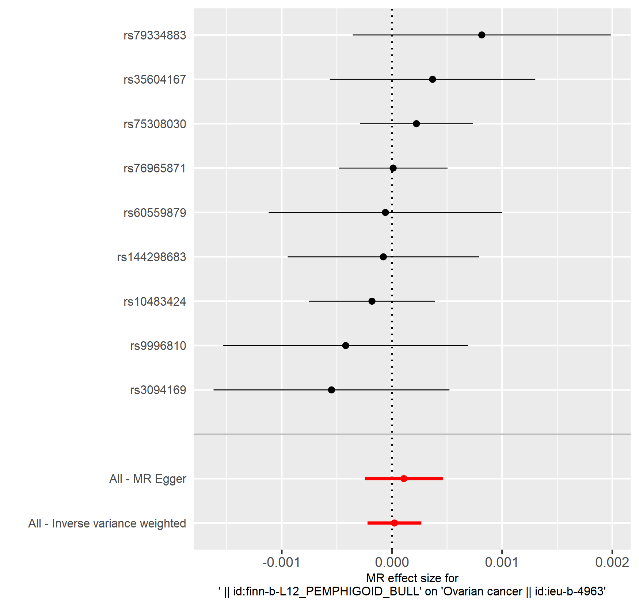

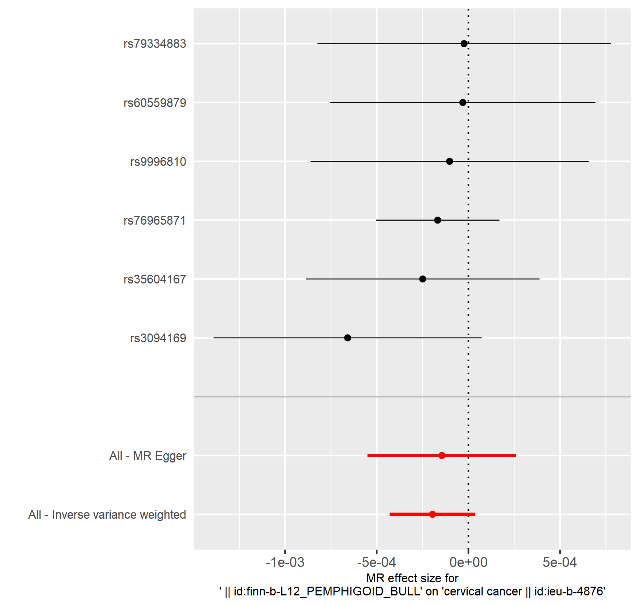


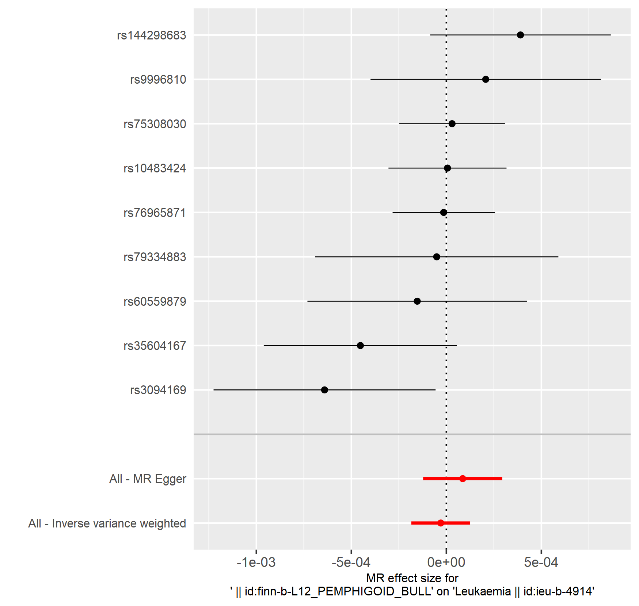

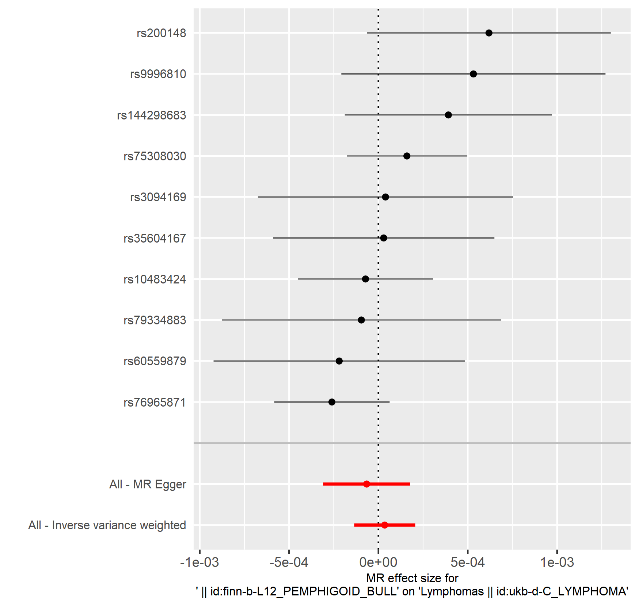

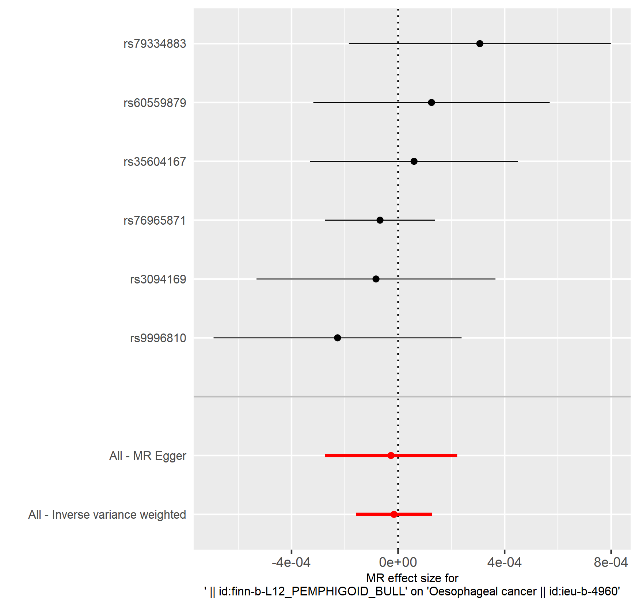


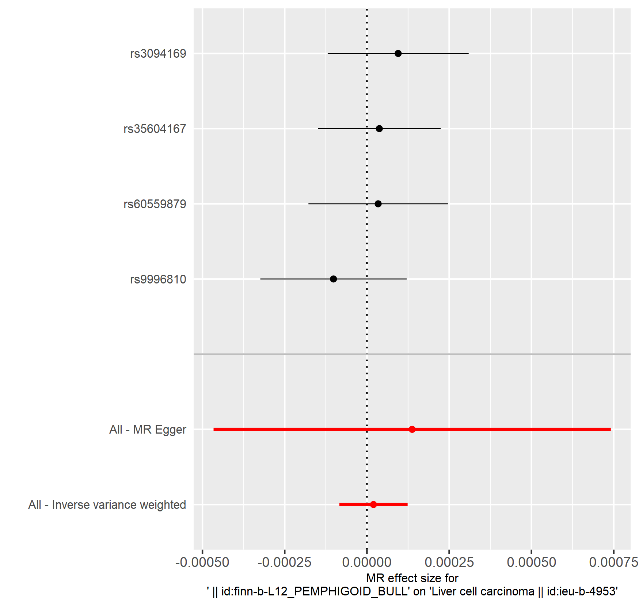


**Supplementary Figure 2** The forest plots of the association between BP and 13 types of malignant tumors in MR analysis.


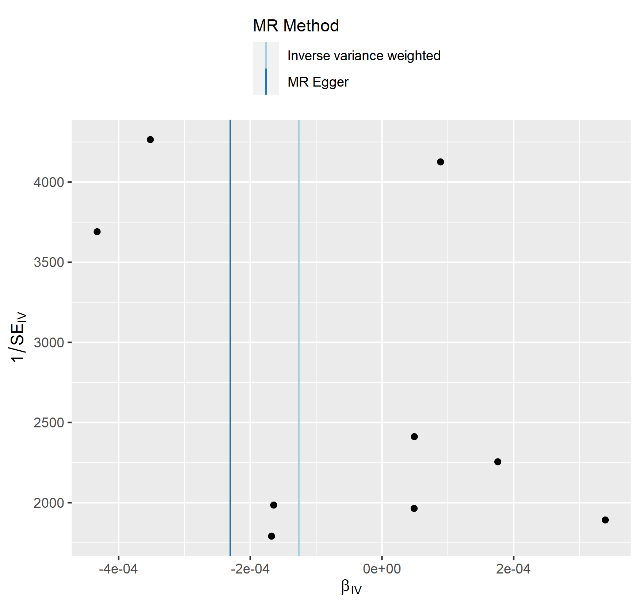

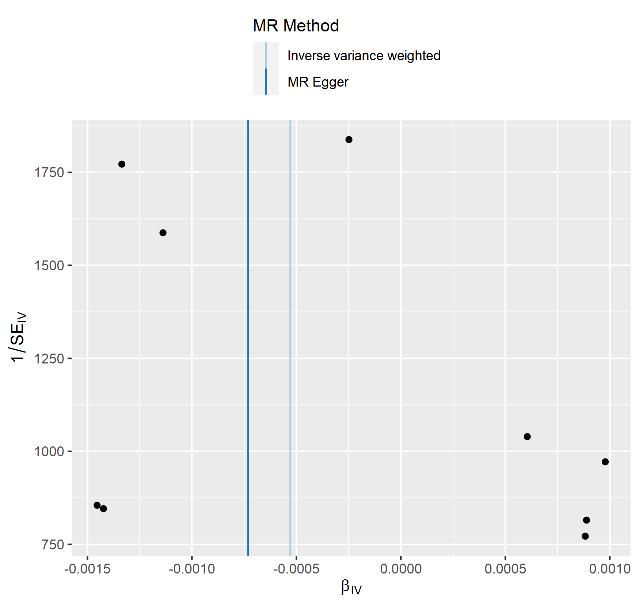

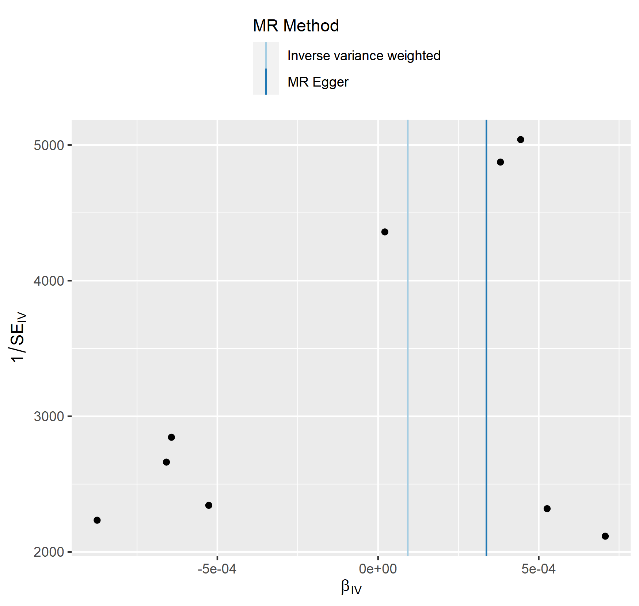


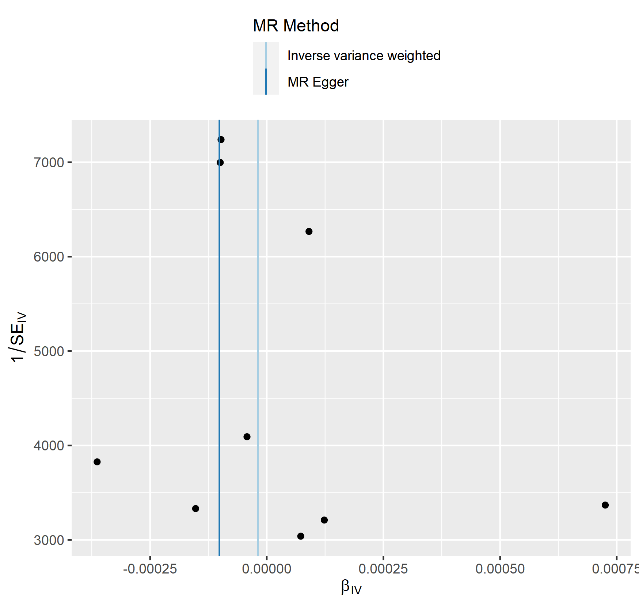

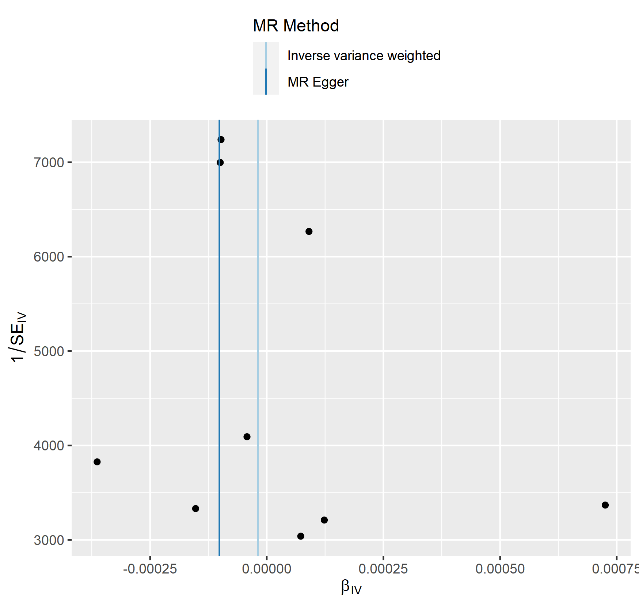

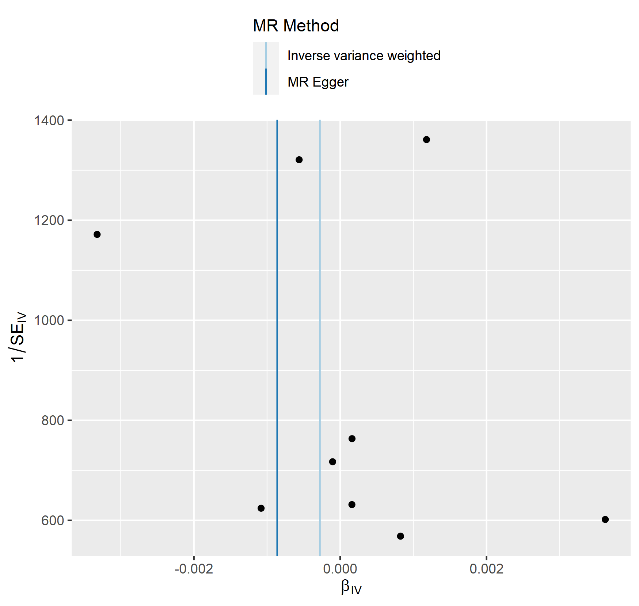


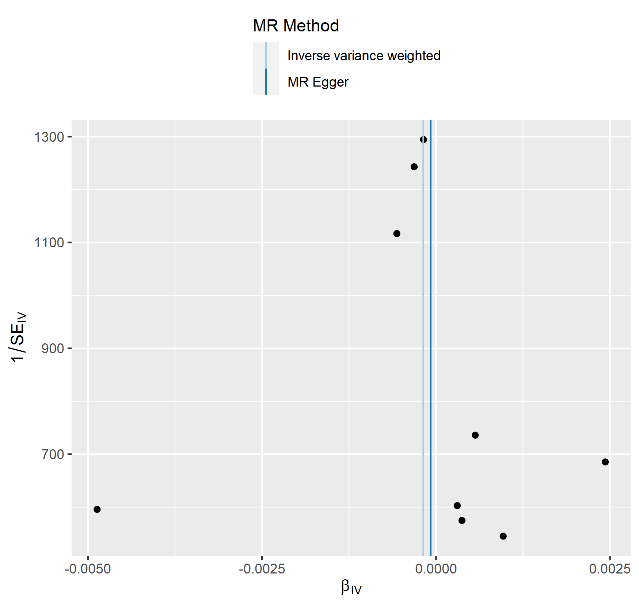

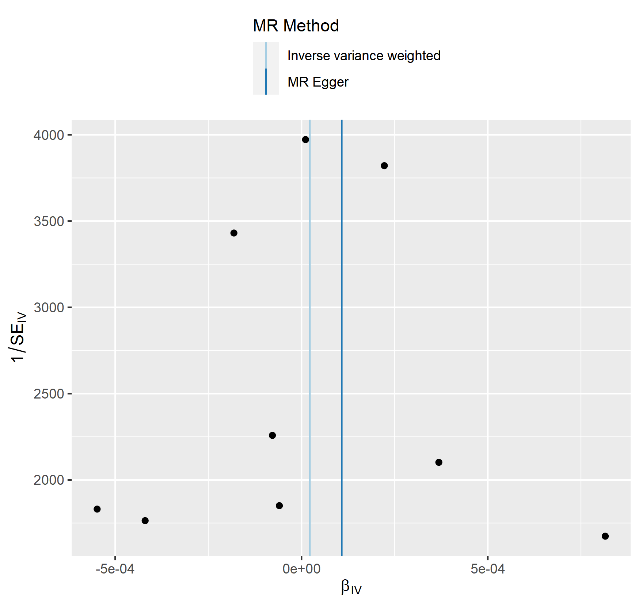

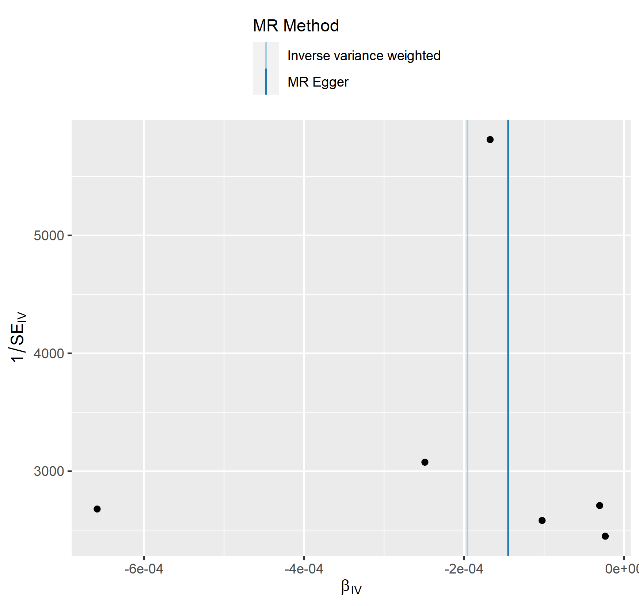


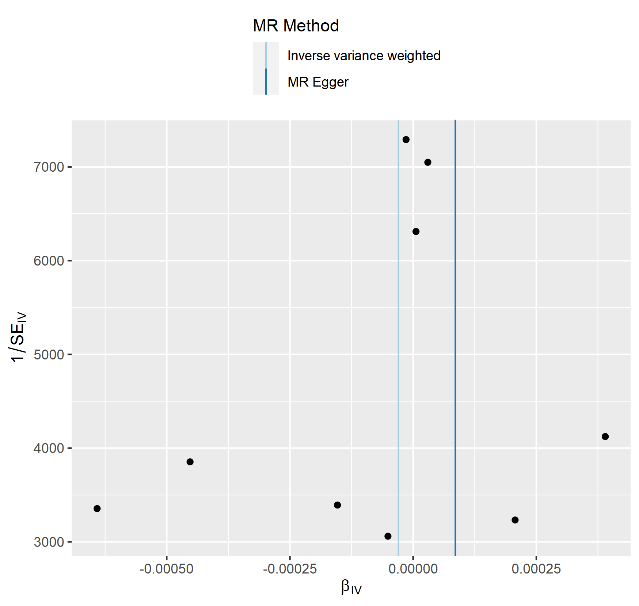

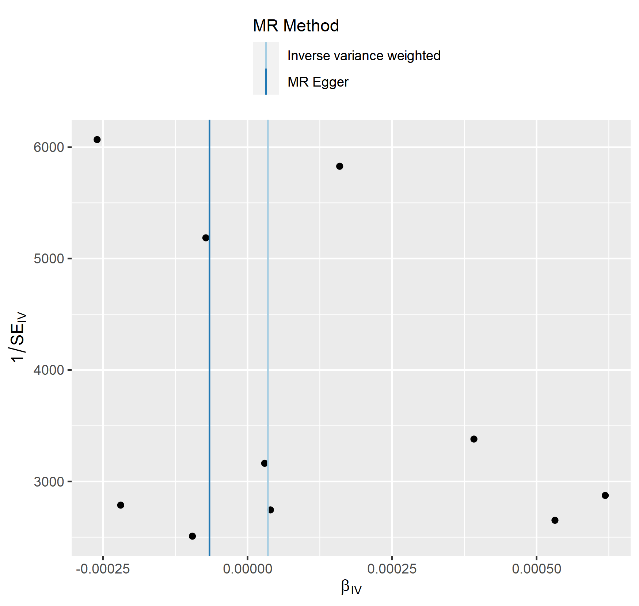

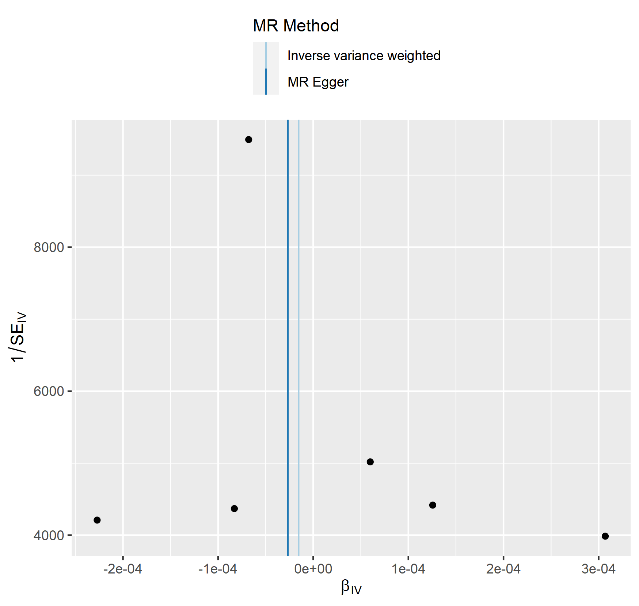


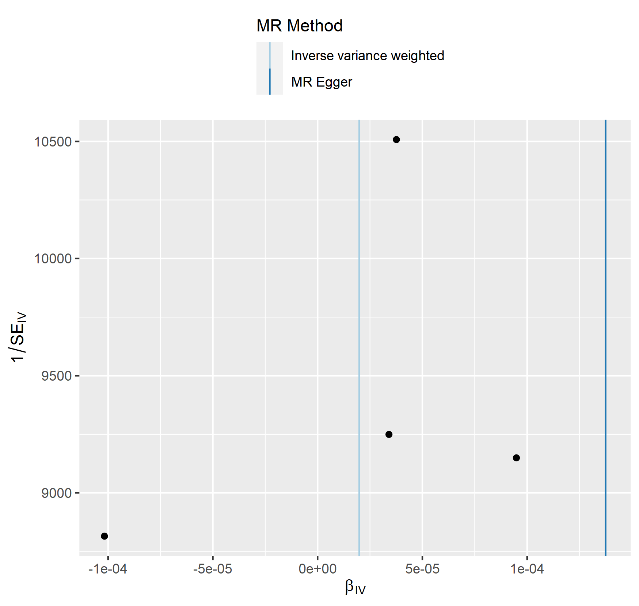


**Supplementary Figure 3** The funnel plots of the association between BP and 13 types of malignant tumors in MR analysis. The arrangement of the Figure is consistent with the preceding ones.


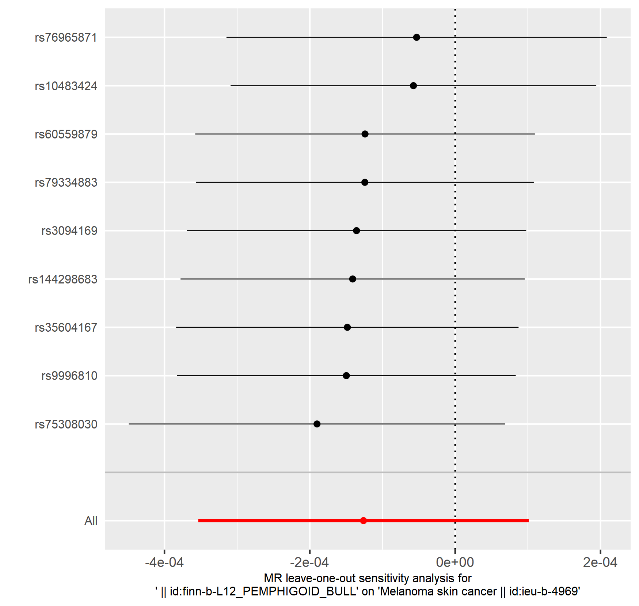

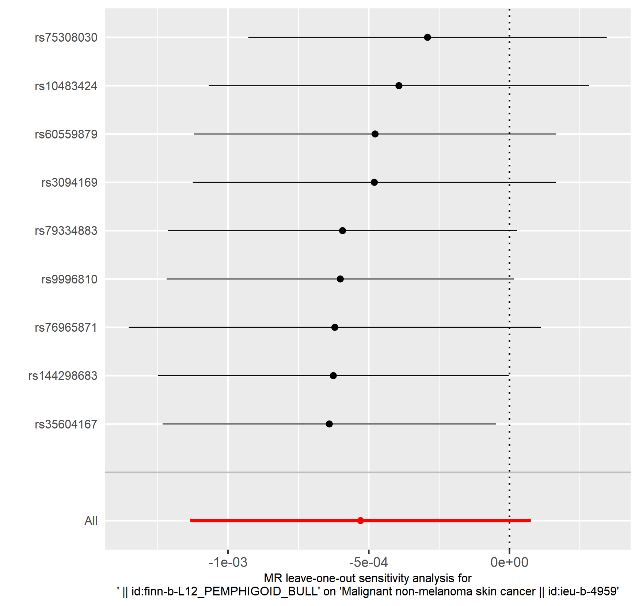

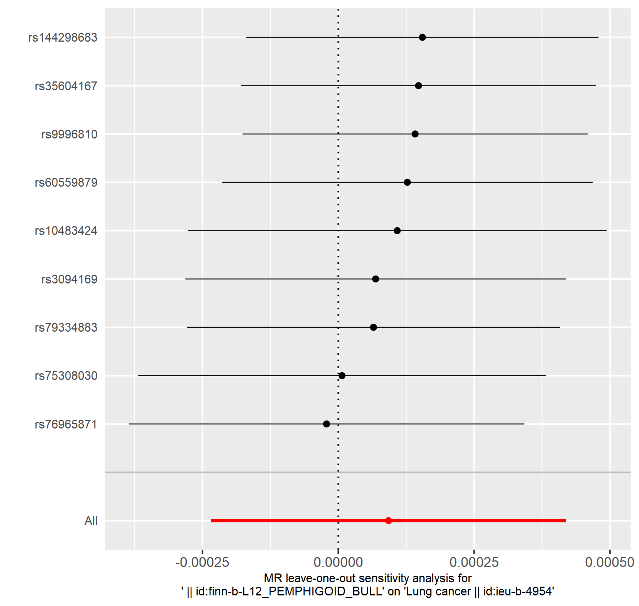


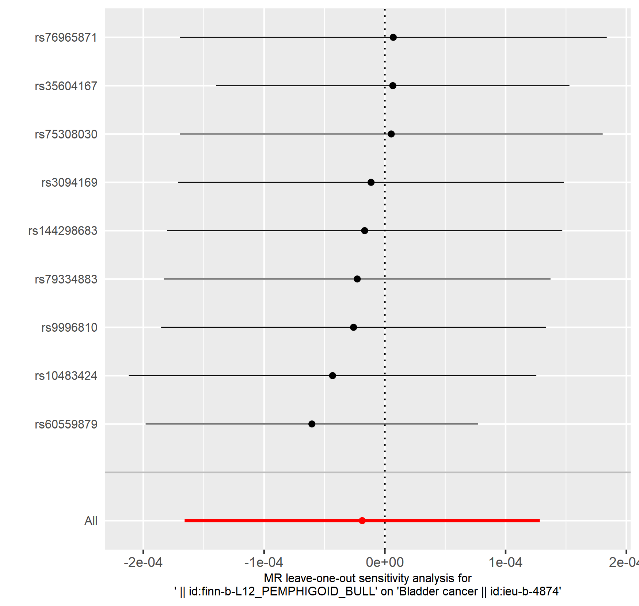

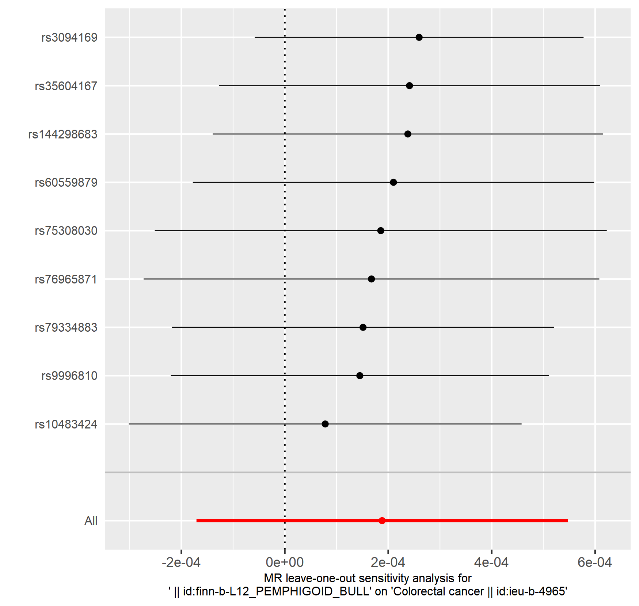

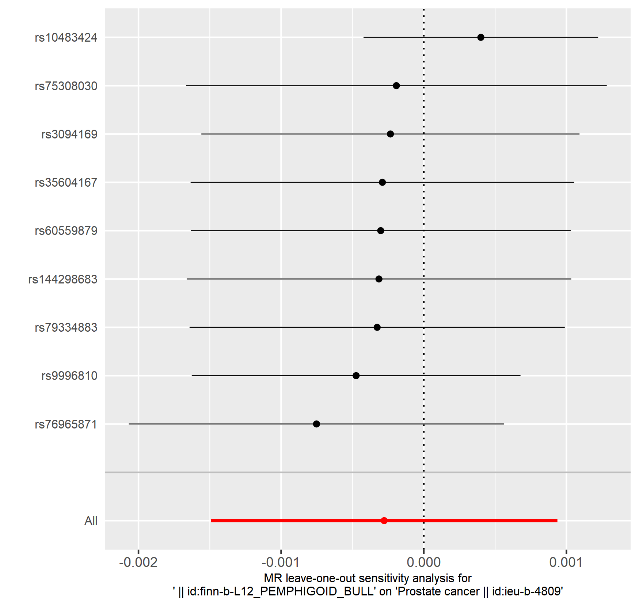


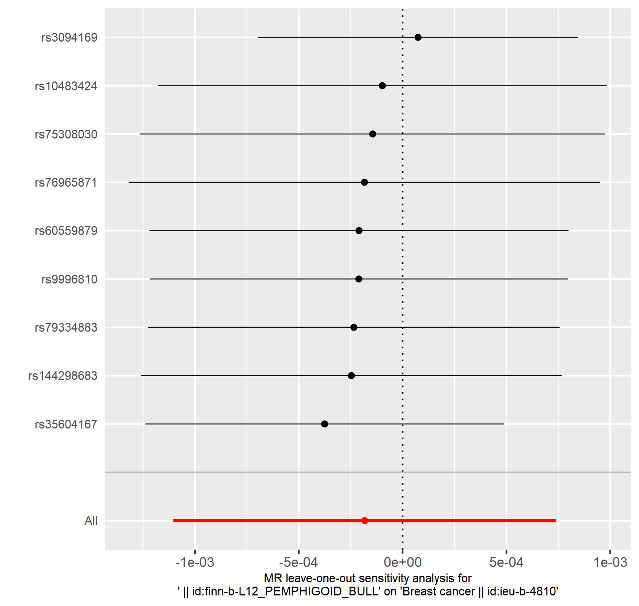

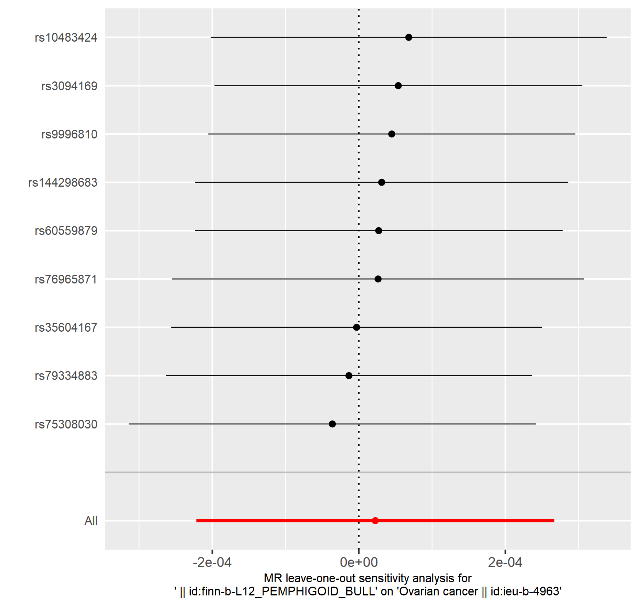

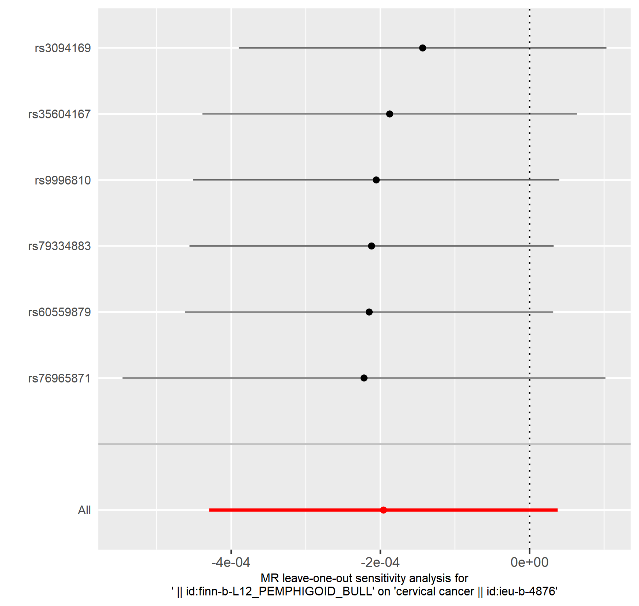


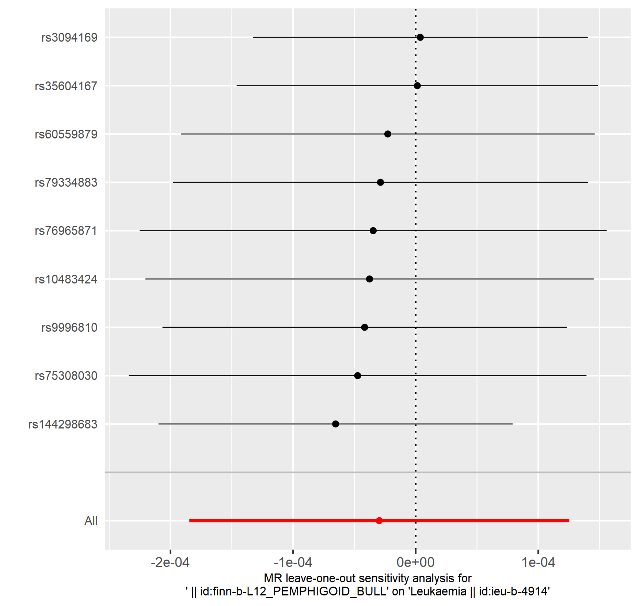

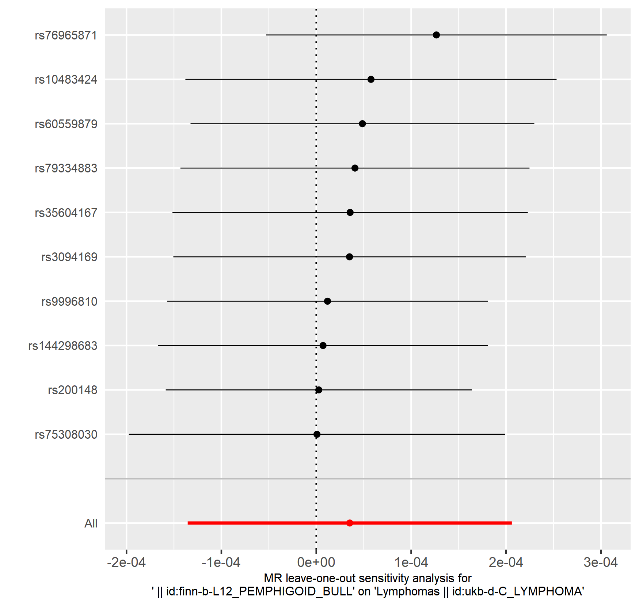

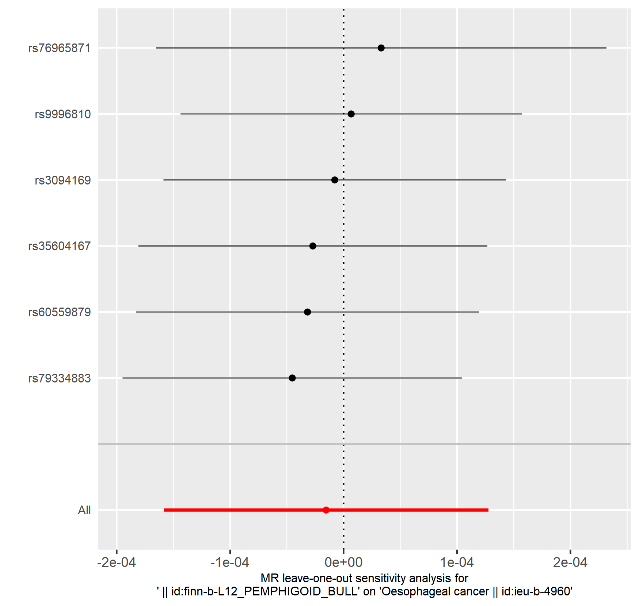


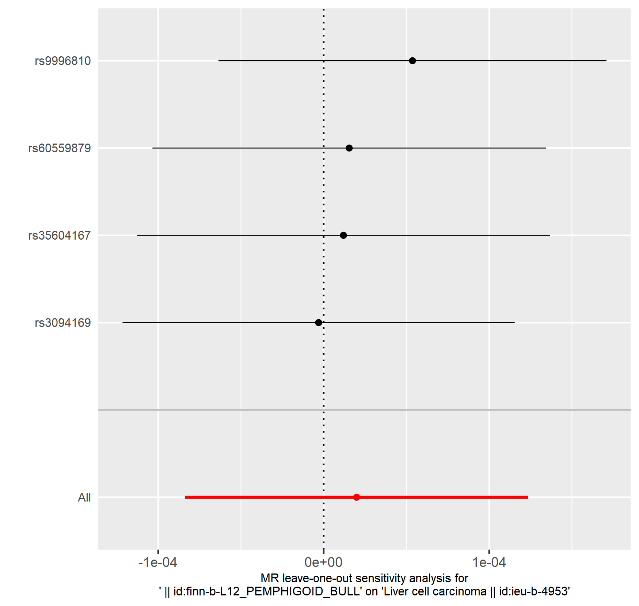


**Supplementary Figure 4** The leave-one-out analysis of the association between BP and 13 types of malignant tumors in MR analysis.
